# Global deposition of potentially toxic metals via faecal material in seabird colonies

**DOI:** 10.1038/s41598-022-26905-5

**Published:** 2022-12-27

**Authors:** Saúl De La Peña-Lastra, Augusto Pérez-Alberti, Tiago O. Ferreira, Miguel Ángel Huerta-Díaz, Xosé L. Otero

**Affiliations:** 1 Department of Functional Biology, Ecology Section, Faculty of Biology, University of Santiago de Compostela, Santiago de Compostela, Spain; 2 CRETUS, Department of Edaphology and Agricultural Chemistry, Faculty of Biology, University of Santiago de Compostela, Santiago de Compostela, Spain; 3 Departamento de Ciencia do Solo, Escola Superior de Agricultura Luiz Queiroz (ESALQ), Universidade de São Paulo, São Paulo, Brazil; 4 Instituto de Investigaciones Oceanológicas, Universidad Autónoma de Baja California, Carretera Transpeninsular Ensenada-Tijuana No. 3917, Fraccionamiento Playitas, C.P. 22860 Ensenada, Baja California Mexico; 5 Estación de Bioloxía Mariña A Graña, REBUSC, Rede de Estacións Biolóxicas da Universidade de Santiago de Compostela, Ferrol, Spain

**Keywords:** Biogeochemistry, Environmental sciences

## Abstract

Seabirds are known to play an important role in the geochemical cycling of macronutrients; however, their role in cycling elements of environmental interest has not been investigated. Guano is an important source of marine-derived nutrients and trace metals in seabird nesting areas, but most of the available information on this topic is derived from local studies. In the present study, we used a bioenergetic model to estimate the amounts of cadmium (Cd), mercury (Hg) and lead (Pb) that are deposited via faecal material in seabird colonies worldwide. The findings showed that the seabirds excreted 39.3 Mg (Mg = metric ton or 1000 kg) of Cd, 35.7 Mg of Hg and 27.2 Mg of Pb annually. These amounts are of the same order of magnitude as those reported for other fluxes considered in the geochemical cycling of these elements (e.g. sea-salt spray, cement production, soil loss to oceans). Most of the deposition occurs in circumpolar zones in both hemispheres and, interestingly, high proportions of the metals in the excrements occur in geochemically labile forms, which can be easily leached into coastal waters and assimilated by marine organisms.

## Introduction

Seabirds are considered biovectors that enable the flow of nutrients across ecosystem boundaries, and they also deposit large amounts of allochthonous debris in their breeding colonies^1,2^. These processes drastically increase the concentrations of nutrients and potentially toxic elements in soils, water, plants and the atmosphere^3–5^. However, most studies carried out to date on this subject have only considered the environmental effects at local geographical scales^6–8^.

Recent studies have demonstrated that seabirds may affect the cycling of some elements at regional and even global scales. Riddick et al.^9^ established that seabird breeding colonies are the main source of ammonia (NH_3_) emissions to the atmosphere in remote locations and that these emissions may greatly affect ecosystems inside and outside of the colonies^4,9^. Otero et al.^10^ estimated that via excretions seabirds deposit 591 Gg of nitrogen (N) and 99 Gg of phosphorus (P) annually in their colonies worldwide. These values are of a similar magnitude as other fluxes usually considered in the global cycling of N and P (i.e. lightning, fishing activities, inorganic P flux from rivers to oceans). To date, however, the amounts of toxic metals that seabirds mobilize from oceanic waters to land have not been estimated.

Considering the important effects that toxic elements may have on aquatic ecosystems^11–13^, we estimated the amounts of cadmium (Cd), mercury (Hg) and lead (Pb) that are excreted via faeces in seabird breeding colonies worldwide^14^. First, we obtained the most up-to-date information on seabird populations from global seabird organizations (e.g. Birdlife International and Wetlands International). We then adapted a bioenergetic model previously applied to N and P ^4,10^ to estimate the amounts of the different elements excreted by seabirds. Finally, we considered the importance of seabirds in the global cycling of Cd, Hg and Pb relative to their main compartments as well as the fluxes between these compartments at the global scale.

## Results and discussion

The estimated total amounts of potentially toxic metals deposited annually through excrements in seabird colonies, considering only breeding seabirds, were 39.3 Mg (1 Mg = 1 metric ton or 1000 kg) of Cd, 35.7 Mg of Hg and 27.2 Mg of Pb (Tables 1; Table S1). However, when the total worldwide population of seabirds was considered (i.e. breeding and non-breeding birds) over the entire annual period, the inputs increased substantially, reaching values of 249 Mg of Cd year^−1^, 226 Mg of Hg year^−1^ and 172 Mg of Pb year^−1^. However, these fluxes should be considered with caution, as most seabird species do not remain in their colonies outside of the breeding season^10^.

**Table 1:** Annual mass (Mg year^−1^) and percentage (in parenthesis) of potentially toxic metal excreted by breeding birds and chicks in the colonies by seabird groups.

Seabird order	Breeding birds and chicks (millions)	Cd	Hg	Pb
		Mg year^−1^ (%)		
Charadriiformes	267	21.7 (55.2)	0.79 (2.2)	22.2 (81.4)
Pelecaniformes	29	3.71 (9.4)	25.8 (72.4)	1.01 (3.7)
Procellariiformes	391	8.47 (21.5)	8.19 (22.9)	1.58 (5.8)
Sphenisciformes	58	5.4 (13.8)	0.87 (2.4)	2.48 (9.1)
Total	745	39.3	35.7	27.2

**Figure 1:**
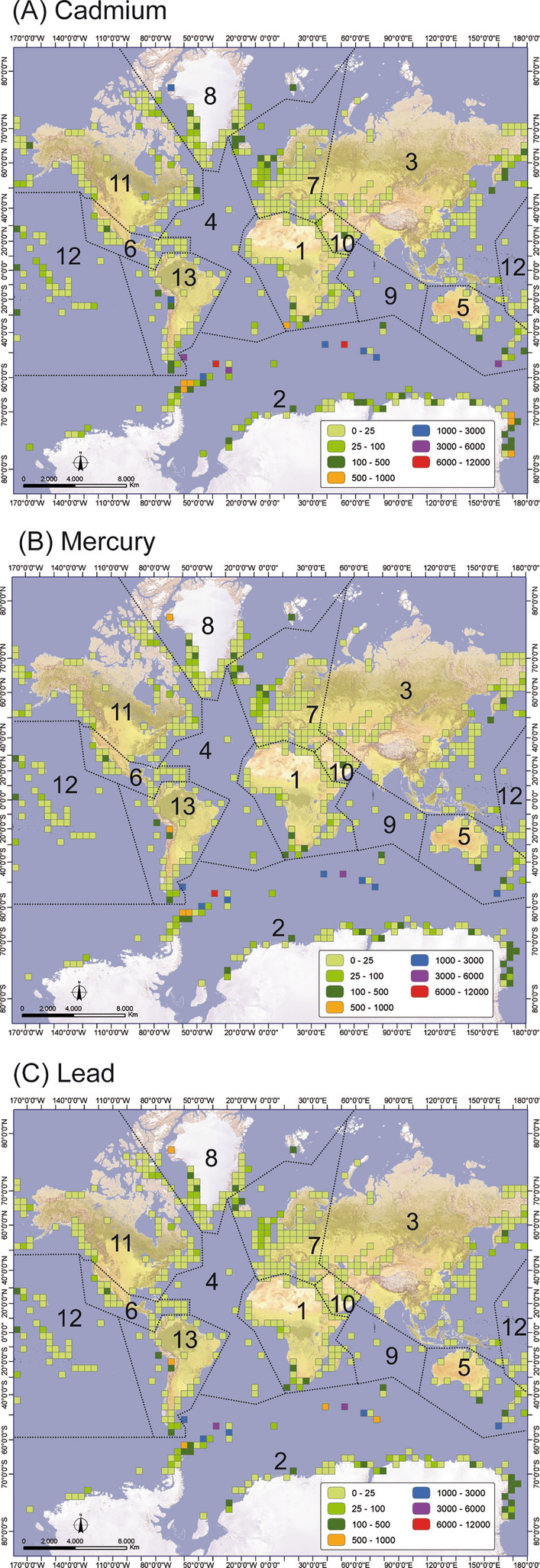
Global distribution of potentially toxic metals excreted by seabird colonies. The distribution of the colonies was arranged as a network grid of 500 km × 500 km square cells and the total number of seabirds in each cell was then estimated. Dashed lines delineate regional boundaries after previous works (Riddick et al. ^4^, Otero et al.^10^): 1. Africa, 2. Antarctica & Southern Ocean, 3. Asia, 4. Atlantic, 5. Australasia, 6. Caribbean & Central America, 7. Europe, 8. Greenland & Svalbard, 9. Indian Ocean, 10. Middle East, 11. North America, 12. Pacific and 13. South America. (see also Table 2). Scale colours show flux ranges in kg year^−1^).

The biogeographical distribution of seabirds is not homogeneous across the world, thus greatly affecting the global deposition of potentially toxic elements (Fig. 1). The present results show that most of the elemental deposition occurs in the polar and subpolar regions of the southern hemisphere. Deposition of toxic metals mainly occurs in the Antarctic and the Southern Ocean, with total amounts of 31.3 Mg Cd year^−1^, 28.4 Mg Hg year^−1^ and 21.6 Mg Pb year^−1^, representing almost 80% of the total metal deposited in seabird colonies worldwide. The next most important areas are Greenland and Svalbard, Australasia, Pacific and South America, representing between 2 and 5% of the total toxic metal deposited in the colonies worldwide (Table 2).

**Table 2:** Seabird population and total amounts of potentially toxic metals excreted in different geographical areas.

Geographical area	Population of breeders and chicks	Cd	Hg	Pb
	(millions)	(Mg year^−1^)		
1. Africa	5.71	0.36	0.33	0.25
2. Antarctica and Southern Ocean	197	31.3	28.4	21.6
3. Asia	37.1	0.55	0.50	0.38
4. Atlantic	0.29	0.07	0.07	0.05
5. Australasia	88.5	1.79	1.62	1.24
6. Caribbean and Central America	2.86	0.26	0.23	0.18
7. Europe	28.3	0.56	0.52	0.39
8. Greenland and Svalbard	194	2.15	1.95	1.49
9. Indian Ocean	11.4	0.18	0.17	0.13
10. Middle East	1.14	0.11	0.10	0.08
11. North America	68.5	0.51	0.46	0.35
12. Pacific	98.5	0.77	0.70	0.53
13. South America	11.7	0.73	0.66	0.50

**Figure 2:**
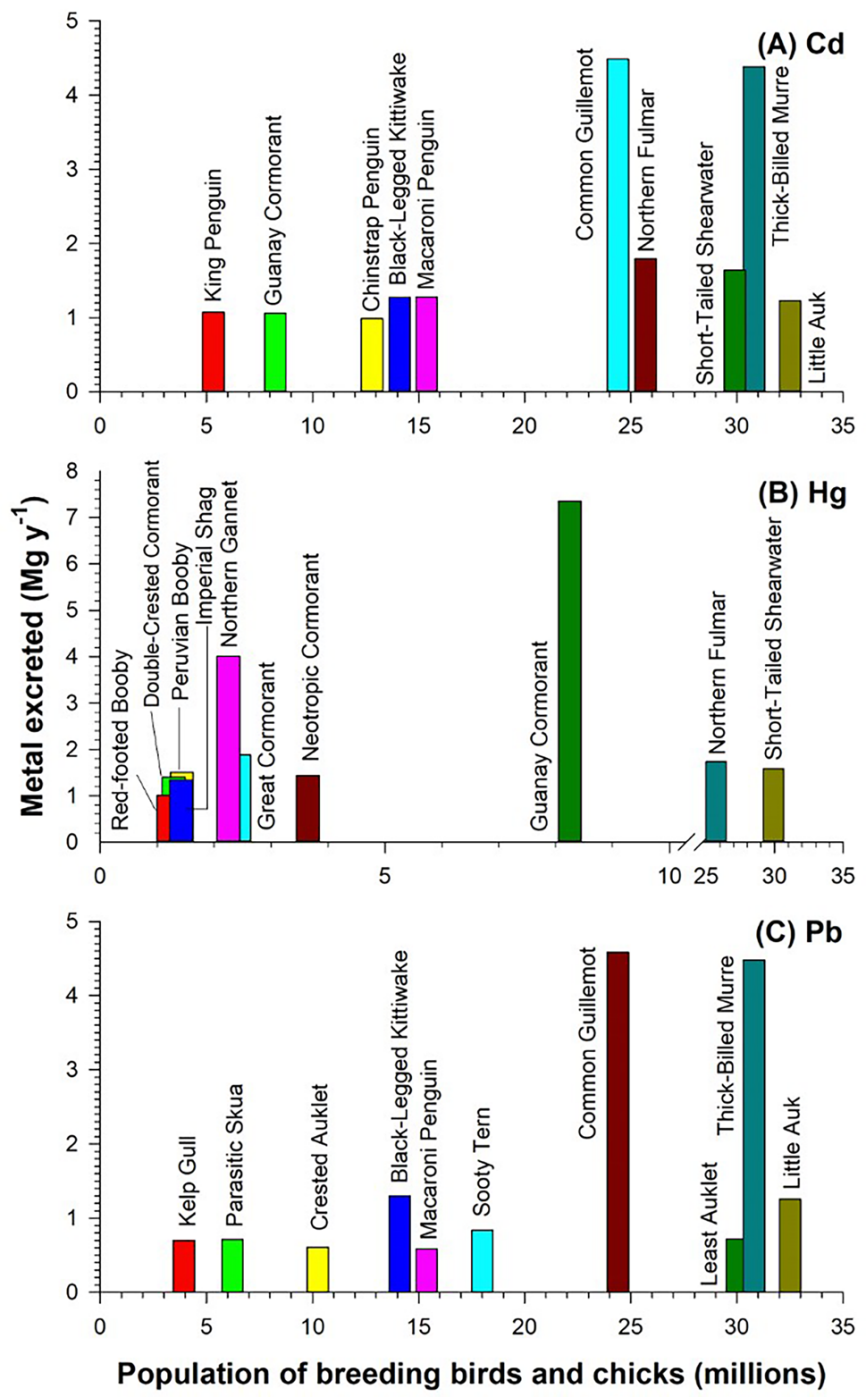
Seabird species excreting the highest amounts of toxic metals on a global scale relative to population size. The importance of seabird species in terms of Cd, Hg and Pb fluxes from marine to continental environments (breeding colonies) depends on various factors, such as population size, body weight, length of the breeding season and type of diet. The contributions of different species are very heterogeneous: penguins are the main contributors of the three toxic metals, mainly because of their large body mass (height: 70–130 cm; weight: 5–40 kg) and the long period of time that they remain in the colony (more than one year), whereas the contributions of smaller species, such as the common guillemot, northern fulmar, short-tailed shearwater and the thick-billed murre, are due to their large population sizes. The separate values for adults and chicks of the 5 species that contribute most of the global excretion of each metal are represented in Table S4.

We found that the order Charadriiformes (which includes seagulls, terns, skuas and auks) contributed most of the Cd (21.7 Mg year^−1^) and Pb (22.2 Mg year^−1^), whereas the order Pelecaniformes (which includes pelicans, gannets and cormorants) contributed most of the Hg (25.8 Mg year^−1^) (Table 1). These findings contrast with those obtained for N and P depositions, which are mainly associated with the Sphenisciformes (penguins), owing to the importance that the bioenergetic model gives to the body weight of each species^10^. In addition, these findings are also consistent with the transfer and accumulation of heavy metals in the tissues of seabirds, mechanisms which are closely related to the diet or position of each species in the trophic chain^15^. For example, the albatross, a pelagic species that mainly feeds on squid, has high Cd tissue concentrations^16,17^, even though it lives far from anthropogenically influenced environments. Other pelagic species that feed on a mixed diet of squid and fish have high tissue concentrations of Hg^15,18^. It has also been shown that top predators (e.g. terns, gulls, and auks) have higher hepatic concentrations of toxic elements than seabirds that occupy lower positions in the marine food chain, such as penguins^3,19^. Finally, the fact that many species in the order Charadriiformes inhabit coastal areas, which are often strongly affected by anthropogenic activities, may also help to explain the importance of this order on a global scale^20–22^. The species of Charadriiformes found to contribute most to the deposition of Cd and Pb were the common guillemot (*Uria aalge*, 3.38 Mg Cd year^−1^, 3.31 Mg Pb year^−1^) and the thick-billed murre (*Uria lomvia*, 3.20 Mg Cd year^−1^, 3.13 Mg Pb year^−1^), whereas those that deposited most Hg were the Guanay cormorant (*Phalacrocorax bougainvilliorum*, 3.35 Mg year^−1^) and the northern gannet (*Morus bassanus*, 2.93 Mg Hg year^−1^) (Fig. 2; Tables 2, Table S3).

The second highest contributions of Cd and Pb to the environment were associated with members of the order Sphenisciformes, which excreted 5.40 Mg Cd year^−1^ and 2.48 Mg Pb year^−1^, with the species contributing the most being the Macaroni penguin (*Eudyptes chrysolophus*, 1.05 Mg Cd year^−1^ and 0.48 Mg Pb year^−1^) and king penguin (*Aptenodytes patagonicus*, 0.90 Mg Cd year^−1^ and 0.48 Mg Pb year^−1^) (Tables S1, S4). Regarding Hg, the order Procellariiformes was the second most important, excreting 8.19 Mg Hg year^−1^, with particularly high contributions from the northern fulmar (*Fulmarus glacialis*; 1.79 Hg Mg year^−1^) and the short-tailed shearwater (*Ardenna tenuirostris*; 1.58 Mg Hg year^−1^) (Tables S1, S3). The individual species of breeding seabirds that contributed the highest amounts of the elements considered were penguins, albatrosses, sheathbills, skuas, pelicans, gannets and cormorants (Tables S1, S4). For example, the Northern Gannet was the second highest depositor of Hg per individual species, which can be attributed to the long periods that these birds spend in the colonies (~ 243 days per year^44^) (Fig. 2). However, chicks accounted for less than ~ 15% of the amounts of elements deposited by adult breeding birds (Table S4). The species accounting for most of the element depositions include four species of cormorant and two species each of seagulls, gannets and pelicans. Among young birds, the Guanay cormorant, thick-billed murre, common guillemot and black-legged kittiwake are particularly important, as these species excrete between 17 and 32% of the total depositions of the elements considered. This can be explained by the large size of these species (e.g. the mean weight of the Guanay cormorant is 2.1 kg), the time they spend in the colonies (e.g. Guanay cormorant, 213 days) and the high reproductive success (e.g. the Guanay cormorant produces 2.4 chicks pair^−1^ year^−1^).

Reported magnitudes of fluxes between different geochemical compartments show that the amounts of metals mobilized by seabirds are important in terms of the global biogeochemical cycling of each element (Tables S5–S7). For example, the flow of Cd between marine and terrestrial environments attributed to seabirds (39 Mg Cd year^−1^; total population 249 Cd Mg year^−1^; Table 1, Table S5) is of the same order of magnitude (or even higher) than that mobilized by other processes, such as sea-salt spray (2–100 Mg Cd year^−1^), wildfires (100–600 Mg Cd year^−1^), windborne dust (200–210 Mg Cd year^−1^) and cement production (64 Mg Cd year^−1^). For Pb, the fluxes from marine to terrestrial environments attributed to seabirds (breeding birds: 27 Pb Mg year^−1^; total population: 172 Mg Pb year^−1^; Table 1, Table S6) are of a similar magnitude to fluxes caused by cement production (268 Mg Pb year^−1^) or sea-salt spray (100–1400 Mg Pb year^−1^). Likewise, the Hg flow associated with seabirds (breeding seabirds: 36 Mg Hg year^−1^; total population: 226 Mg Hg year^−1^; Table 1, Table S7) is of the same order of magnitude as that associated with soil loss to the oceans (200–380 Mg Hg year^−1^) or with sediment accumulation in deep oceanic sediments (200–600 Mg Hg year^−1^).

**Figure 3:**
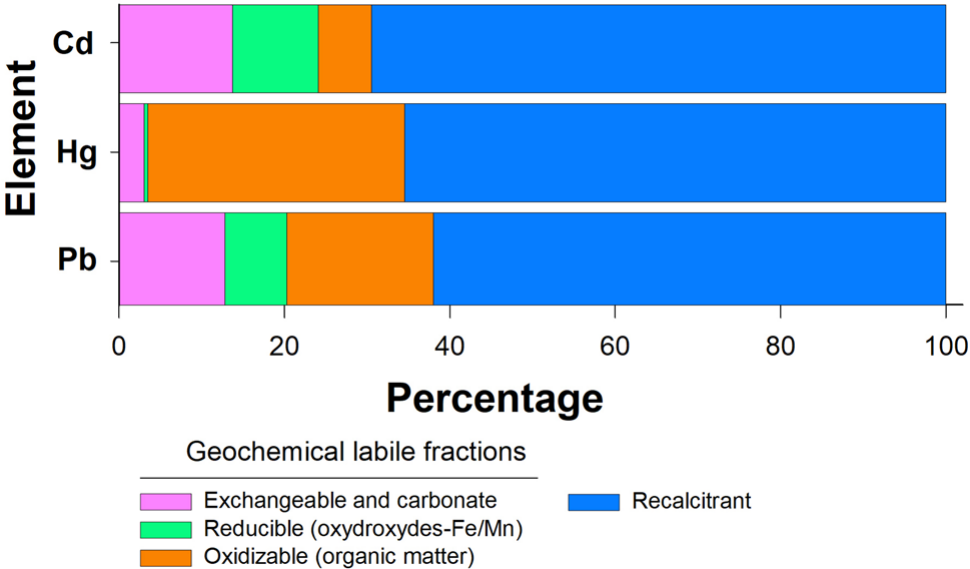
Fractionation of potentially toxic elements in different geochemical labile and recalcitrant fractions measured in yellow-legged gull (*Larus michahellis*) droppings (n = 10).

The above findings demonstrate the importance of seabirds in the transfer of contaminants from marine environments to land and indicate that these inputs should therefore be included in the between-compartment fluxes that determine the global biogeochemical cycling of each of these three elements. It must also be considered that, unlike other more diffuse flows (e.g. atmospheric emissions, atmospheric deposition), the deposition of toxic elements by seabirds is generally concentrated in specific areas of the coast, and that the metals generally occur as labile geochemical forms that can readily move towards shallow coastal waters^5^. For example, a small colony of yellow-legged gulls in the NW Iberian Peninsula (~ 16,800 individuals occupying an area of 100 ha) deposits 700 g of Cd, 100 g of Hg and 360 g of Pb every year^25^. Geochemical analysis of bird excrements shows that ~ 35% of the total element content present in these phases is associated with the labile forms (Fig. 3).

More specifically, labile forms of Hg are mainly associated with organic matter and/or absorbed to soil colloids, whereas Pb and Cd are associated with Fe oxyhydroxides (Fig. 3; Table S8). In the colonies, where vegetation is typically sparse as a result of the birds trampling the soil, the excrements are eroded and transported via run-off towards coastal waters. Metals adsorbed to organic matter and clays (fraction F1) may be desorbed under conditions of high salinity, whereas the metal fraction associated with Fe/Mn oxyhydroxides (fraction F2) could eventually be released to the water by dissolution of the oxyhydroxides in reduced environments ^10,23,24^. Both processes will favour the bioavailability of all three elements (see Otero et al.^10^). Leaching of metals towards coastal waters may be particularly important in remote, pristine areas, especially in the Antarctic and Southern Ocean regions, where most seabird species occur (Fig. 1). Furthermore, increasing rainfall levels in the polar zones^25^ and the accelerated melting of frost and permafrost due to increasing temperatures associated with climatic change may mobilize metals accumulated in the guano and soils in polar and subpolar regions^26–29^. The results of this study highlight the seabird-driven connectivity of Cd, Pb and Hg between terrestrial and marine ecosystems, especially in polar regions, where the effects of these birds are more pronounced. The accelerated warming of the oceans is increasing run-off in the coastal waters of these regions, thereby affecting the biogeochemical processes that regulate trace metal fluxes of environmental concern and, consequently, their influence on the food chain^30,31^. Our findings can help improve predictions based on biogeochemical models, allowing for a better understanding of the consequences of climate change in polar waters.

## Methods

### Estimation of the worldwide seabird population

Seabirds usually gather in large colonies during the breeding season and spend most of their lives between the sea and the areas around the colonies^32,33^. To estimate the seabird populations, we first updated the information included in Otero et al.^10^ by consulting articles and data reported by international organizations (e.g. BirdLife International, for further details see also Otero et al.^10^). In a similar way as in the previous study^10^ and in order to estimate the global seabird population, we used the available data on breeding pairs, adding another 30% to represent non-breeding birds, as reported by other authors^34,35^. The bird species (323 species), size of the breeding population and the source of information used are included in Table S1. The seabirds were grouped into the following orders for the sake of simplicity: Sphenisciformes (penguins), Procellariiformes (albatrosses, shearwaters and petrels), Pelecaniformes (pelicans, boobies, frigatebirds, tropicbirds and cormorants) and Charadriiformes (gulls, terns, guillemots and auks).

The global population of breeding seabirds and chicks was estimated to be 745 million individuals (Table S1). After addition of the estimated number of non-breeding birds (30%^4,36,37^), the total seabird population was estimated to be 969 million individuals. This figure is lower than reported in previous studies, in which estimates vary between 900 and 1180 million^4,10,36^. However, the difference is consistent with the overall decline in the populations of many seabird species attributed to the effects of global climate change on the availability of food. For example, the population of the chinstrap penguin (*Pygoscelis antarcticus*) is declining at an alarming rate due to global climate perturbations at the regional level affecting the sea ice phenology, a process that has repercussions for penguin food supply and competition for resources^37,38^.

Regarding the different orders, the most numerous are the Procellariiformes, with 391 million individuals and 123 species, followed by the Sphenisciformes, with 58 million individuals and 17 species, and the Pelecaniformes, with 29 million individuals and 53 species (Table S1). The most numerous species worldwide are the Antarctic prion (*Pachyptila desolata*, 62.5 million individuals), the little auk (*Alle alle*, 32.5 million), the thick-billed murre (*Uria lomvia*, 30.8 million), the leak auklet (*Aethia pusilla*, 30 million), the short-tailed shearwater (*Ardenna tenuirostris*, 29.9 million), the northern fulmar (*Fulmarus glacialis*, 25.7 million) and the sooty shearwater (*Ardenna grisea*, 25.5 million) (Table S1).

Mapping the global distribution showed that seabird colonies are mainly located in polar regions, with more than half of the total population concentrated in the Antarctic and the subantarctic islands (197 million) and in Greenland and the Svalbard islands (194 million). However, as previously pointed out ^10^, although the numbers of seabirds are similar in both polar regions, the amounts of elements excreted are not necessarily similar. The number of individuals, the body weight of the species and the duration of the breeding season are the main reasons why greater amounts of elements are deposited by seabirds in the Antarctic and the subantarctic islands than in other parts of the world. However, some species that inhabit other areas, such as industrial zones, are subjected to high levels of exposure and contamination.

### Global excretion of Cd, Hg and Pb by adult breeding birds

The global amounts of potentially toxic metals excreted by breeding seabirds (E_excr(br)_: Cd_excr(br)_, Hg_excr(br)_ or Pb_excr(br)_) were calculated using Eq. (1), which represents a modified version of the bioenergetic models proposed by Otero et al.^10^.

1 $${E}_{excr (br)}=\frac{9.2.{M}^{0.774}}{{F}_{Ec}.{A}_{eff}}\cdot {F}_{E}\cdot {t}_{breeding}\cdot {f}_{tc}$$

The variables included in this model were the amount of each metal (F_E_, g Cd, Hg or Pb g^−1^ wet weight; Table S2) excreted by the adult biomass of each bird (M, in g bird^−1^), the energy content of the foodstuff (F_Ec_, in kJ g^−1^ wet weight), the efficiency of assimilation of the metals ingested (A_eff_, in kJ [energy obtained] kJ^−1^ [energy in the foodstuff]), the duration of the breeding season (t_breeding_, in days) and the proportion of time spent in the colony during the breeding season (f_tc_, a dimensionless parameter):

The data reported by Otero et al.^10^ were used to calculate t_breeding_ and f_tc_, while the mean energy contents (F_Ec_ = 6.5 kJ g^−1^) of the seabird diets used in Eq. (1) have been reported in different studies^4,10,39^. F_E_ represents the quantity of metal excreted by each seabird species (g of Cd, Hg or Pb, Table S2), calculated by using the metal content in faecal material, reported in the references compiled in Table S3, relative to the theoretical nitrogen content (0.036 g N g^−1^)^40^. The term A_eff_ represents the efficiency of conversion of the energy from foodstuff consumed (kJ obtained for the bird for each kJ consumed), with an assumed mean value of 0.8^4,10,39,40^ (for more details on the calculation and uncertainties of the method, see supporting information).

### Global excretion of Cd, Hg and Pb by chicks

The amounts of Cd, Hg or Pb excreted annually by chicks (E_excr(ch)_: Cd_excr(ch)_, Hg_excr(ch)_ or Pb_excr(ch)_ respectively) was estimated using Eq. (2):

2 $${E}_{excr(ch)}=\frac{28.43 {M}_{fledging}^{1.06}}{{F}_{Ec}.{A}_{eff}}\cdot {F}_{E}\cdot {t}_{breeding}\cdot {f}_{tc}\cdot \frac{{P}_{chicks}}{2}$$

Chick attendance in the colony is estimated as the length of time between hatching and fledging. The annual amounts of the metals excreted by the chicks (g metal bird^−1^ year^−1^ in the colony, where metal = Cd, Hg or Pb; Eq. 2) were estimated from the mean weight of the chicks at fledging (M_fledging_, g bird^−1^) and the breeding productivity (P_chicks_, chicks fledged per pair). In the same way, P_chicks_ was used to calculate the population of chicks^4,10,39,40^.

The amounts of the toxic metals (Cd, Hg and Pb) excreted by the seabird colonies in the different regions were estimated using the data reported by Otero et al.^10^, which included the locations of the seabird colonies identified by their geographical coordinates and the amounts of N and P excreted. Estimation of the amounts of the metals excreted by seabirds in their colonies worldwide is limited by the scarcity of data on the population sizes, geographical location and number of species per colony. To resolve this problem, we used the amount of N produced by each colony, which is proportional to the number of seabirds in each location^10^. First, we calculated the proportions (by weight) of N:metal excreted by each seabird species. We then used these values to estimate the amount of metal excreted by each colony.

### Map construction

In order to calculate the amounts of toxic metals excreted by seabird colonies in different regions, we used the supplementary data provided by Riddick et al.^4^. Once the amounts of each metal excreted (in kg) in each colony was calculated, maps were constructed using the Create Fishnet tool in ArcGis 10.8.2 (ESRI, License USC, Santiago de Compostela University; https://www.usc.gal/gl/servizos/atic/software/catalogo/) to generate a network of square cells of side 500 km. The Union tool was used to match more than 3000 colonies with each cell. The Dissolve tool was used to group the cells and also to calculate the number of points in each cell and the total amounts of each metal. Finally, the Field Calculator tool was used to calculate the density of each metal in each cell by dividing the area of each cell by the previous sum.

### Extraction of potentially toxic metals

Toxic metals were extracted from fresh faecal material excreted by Yellow-legged gulls (*Larus michahellis*). The material was obtained directly from the rocks in the yellow-legged gull colonies in the Atlantic Islands of Galicia National Park (NW Spain), without the need to handle live individual birds. Each sample corresponded to a composite sample made up of 5 subsamples of faecal material. The samples were frozen at -32 ºC until required and were lyophilized prior to analysis to prevent loss of elements due to volatization.

The total concentrations of the metals were determined in 0.5 g (dry weight) of the lyophilized faecal material, previously ground in an agate mortar. The metals were extracted from the samples by microwave-assisted acid digestion (9 ml of 14.4 M HNO_3_: 3 ml of 12 M HCl for 25 min). Once extracted, the metals were measured by ICP-OES spectrometry (Perkin Elmer, Optima 4300 DV). Certified soil standards were used to validate the method of trace metal extraction (SRM 2709a, SMR2710a, SRM2711a from NIST, U.S.A.), with a mean recovery rate of > 90%^41,42^.

Sequential extraction was also carried out in the fresh faecal material, using the method proposed by the Community Bureau of Reference (BCR), which enables separation of metals into three geochemically reactive fractions as follows^43,44^:

- Fraction F1 (acid-soluble fraction), extracted from 1 g of faecal material with 40 ml of 0.11 M CH_3_COOH, shaking for 16 h at room temperature, followed by centrifugation for 20 min at 8560*g* and 4 °C. This fraction includes exchangeable metals and metals associated with carbonates. These metals are readily released in the form of exchangeable ions as a result of slight changes in environmental conditions (e.g. salinity).
- Fraction F2 (reducible fraction), extracted with 40 ml of 0.5 M NH_2_OH-HC at pH 1.5, shaking for 16 h, followed by centrifugation for 20 min at 8560*g* and 4 °C. This fraction includes trace metals associated with amorphous Fe/Mn oxyhydroxides.
- Fraction F3 (oxidizable fraction), extracted with two 10 ml of H_2_O_2_ 30% at 85 °C for 1 h, followed by addition of 20 ml of 1 M NH_4_COOCH_3_ at pH 2 and shaking for 16 h followed by centrifugation for 20 min at 8560*g* at 4 °C. This extraction represents the fraction of trace metals bound to organic matter.
- Fraction F4 (recalcitrant), calculated as the difference between the total metal concentration of each metal minus the sum of the three reactive fractions [F4 = ∑(F1 ⟶ F3)—total concentration of metal^24–27^. This fraction can be either associated with recalcitrant organic matter or with metals associated with silicates.

For each fraction, the values are expressed relative to the dry weight of the sample, previously determined by drying a subsample at 105 °C. Finally, the total metal content and the distribution of each metal in the different geochemical fractions depend on the diet, which varies between different seabird species and also seasonally within the same species^45^.

## Supplementary Information

Supplementary Information.

## Data Availability

Any correspondence and/or requests for material should be addressed to X.L.O.
