# Supplementary material for: Global deposition of potentially toxic metals via faecal material in seabird colonies
Source: Sci Rep. 2022 Dec 27;12:22392. doi: 10.1038/s41598-022-26905-5 (PMC9794694; doi:10.1038/s41598-022-26905-5)
Supplement: Supplementary file 1 — Supplementary Information. [file 41598_2022_26905_MOESM1_ESM.docx]

**Global deposition of potentially toxic metals via faecal material in seabird colonies**

Saúl De La Peña-Lastra^1^, Augusto Pérez-Alberti^2^, Tiago O. Ferreira^3^, Miguel Ángel Huerta-Díaz^4^, Xosé L. Otero^2,5^

^1^ Department of Functional Biology, Ecology Section, Faculty of Biology, University of Santiago de Compostela; Santiago de Compostela, Spain.

^2^ CRETUS Institute, Department of Edaphology and Agricultural Chemistry, Faculty of Biology, University of Santiago de Compostela; Santiago de Compostela, Spain.

^3^ Departamento de Ciencia do Solo, Escola Superior de Agricultura Luiz Queiroz (ESALQ), Universidade de São Paulo; São Paulo, Brazil.

^4^ Instituto de Investigaciones Oceanológicas, Universidad Autónoma de Baja California, Carretera Transpeninsular Ensenada-Tijuana No. 3917, Fraccionamiento Playitas, C.P. 22860, Ensenada, Baja California, Mexico.

^5^ REBUSC. Rede de Estacións Biolóxicas da Universidade de Santiago de Compostela, Estación de Bioloxía Mariña A Graña; Ferrol, Spain.

| **Content** | **Page** |
| --- | --- |
| Supplementary methods information | S3 |
| Table S1. Seabird population and total amounts of toxic metals excreted…………...…….……. | S6-S13 |
| Table S2. F_E_ factor ………………………….…………………...….……………………………...... | S14 |
| Table S3 Bibliographic compilation of Cd, Hg and Pb content in faecal material of different seabirds……………………………………………..………………………… | S14-S15 |
| Table S4. Cd, Hg and Pb excreted by adults and chicks of that seabird species that contribute the most to deposition ………………………………………………………………..……………….. | S16 |
| Table S5. Global natural and anthropogenic emissions and fluxes of Cd………………….….…. | S17 |
| Table S6. Major compartments of Hg in the Earth’s surface and fluxes……………………..…... | S18 |
| Table S7. Global natural and anthropogenic emissions and fluxes of Pb……..…………………. | S19 |
| Table S8 Total concentration and metal partitioning in faecal material of *Larus* *michahellis* in colonies from NW Spain…..…………………………………………………………………………… | S19 |
| References of supplementary tables……………………………………….………………………… | S20-22 |

1. **Methods information**

**Uncertainty in the input data.** The parameters included in the bioenergetic models are subject to some degree of uncertainty, mainly because of the fact that the behavior of each seabird species depends on the location of the breeding colony, which may change annually. Additionally, the reproductive success of the seabirds can vary greatly from year to year, along with the number of days spent in the colony and may even vary within different populations of the same species^1^. The size of the populations can also vary considerably depending on the type of census method used and on the interannual variations in seabird populations caused by variations in the El Niño-Southern Oscillation (seabirds in Pacific and Antarctic regions) or in the North Atlantic Oscillation^2^. For all of the above reasons, associated errors of 36% for seabird population numbers, 23% for variations in dietary composition and 13% for the presence of non-breeding birds have been proposed^2,3^. However, considering even the highest levels of these uncertainties did not significantly affect the estimates made in the present study, as the amounts of metals mobilized by seabirds remain high relative to other natural and/or anthropogenic processes.

To estimate the amount of each metal excreted, we carried out the following actions:

1. Compilation of all data available for each metal in seabird excrements.
2. Calculation of the mean value of each metal excreted for each order, due to the scarcity of data for some species.
3. Estimation of N:metal ratios using the N data already available for calculating the amount of each metal.
4. Once the estimates were completed for each metal per seabird species, a similar procedure was used to build the distribution maps.

**Example of the calculation of excreted metal (Pb) carried out for *Aethia cristatella*:**

$E_{excr (br)}=\frac{9.2.M^{0.774}}{F_{Ec}.A_{eff}}.F_{E}.t_{breeding}.f_{tc}$ (1)

The data reported by Otero et al. ^3^ were used to calculate t_breeding_ and f_tc_, while the mean energy contents (F_Ec_ = 6.5 kJ g^-1^) of the seabird diets used in Equation 1 have been reported in different studies ^4,5,6,7^. F_E_ is the theoretical content of each metal (g Cd, Hg or Pb), which was obtained for each metal from the references compiled in Table S7 and relative to the theoretical nitrogen content (0.036 g N g^-1^)*^6^*. The term A_eff_ represents the efficiency of conversion of the energy from foodstuff consumed (kJ obtained for the bird for each kJ consumed), with an assumed mean value of 0.8 ^4,5,6,7^.

The equation used to estimate the amounts of Cd, Hg or Pb excreted annually by chicks (E_excr(ch)_: Cd_excr(ch)_, Hg_excr(ch)_ or Pb_excr(ch)_ respectively), was obtained from an expression similar to Equation 1:

$E_{excr(ch)}=\frac{28.43 M_{fledging}^{1.06}}{F_{Ec}.A_{eff}}.F_{E}.t_{breeding}.f_{tc}.\frac{P_{chicks}}{2}$ (2)

Chick attendance in the colony is estimated as the length of time between hatching and fledging. The annual amounts of the metals excreted by the chicks (g Cd bird^-1^ year^-1^ in the colony, g Hg bird^-1^ year^-1^ in the colony or g Pb bird^-1^ y^-1^ in the colony; Equation 2) were estimated from the mean weight of the chicks at fledging (M_fledging_, g bird^-1^) and the breeding productivity (P_chicks_, chicks fledged per pair). In the same way, P_chicks_ was used to calculate the population of chicks ^2,4,5,6^.

Data from Riddick et al.^2^:

| **Species** | **Species** | **M-Adult weight (g)** | **Days of stay in the colony** | **Proportion of time spent in the colony during the breeding season** | **Nº chicken per couple** | **Fledge mass (g)** |
| --- | --- | --- | --- | --- | --- | --- |
| Crested Auklet | *Aethia cristatella* | 260 | 122 | 0.6 | 0,50 | 240 |

Reported mean values of the amounts of metals present in excrement:

|  |  |  |  |  |
| --- | --- | --- | --- | --- |
| **Order** | **N**  **(mg kg^-1^)** | **Pb**  **(mg kg^-1^)** | **Cd**  **(mg kg^-1^)** | **Hg**  **(mg kg^-1^)** |
|  |  |  |  |  |
|  |  |  |  |  |
| Charadriiformes | 67.7x10^3^ | 14.4 | 14.1 | 0.516 |
| Pelecaniformes | 105x10^3^ | 2.11 | 7.76 | 53.9 |
| Procellariiformes | 176x10^3^ | 2.46 | 13.2 | 12.8 |
| Sphenisciformes | 114x10^3^ | 0.924 | 2.03 | 0.325 |
|  |  |  |  |  |

**Calculation:** Correction factor applied in the bioenergetic model for each metal

Data from Table S2

|  |  |  |  |
| --- | --- | --- | --- |
| Order | **Pb** | **Cd** | **Hg** |
|  |  |  |  |
|  |  |  |  |
| Charadriiformes | 7.65x10^-6^ | 7.49x10^-6^ | 2.74x10^-7^ |
|  |  |  |  |

F_E_ Charadriiformes = (Pb/N)*0,036, (Cd/N)*0,036, (Hg/N)*0,036

***Aethia cristatella* (Charadriiformes)**

Pb excreted by adults (g Pb bird y^-1^ in the colony) = (9.2*(260^0,774^)*7.65x10^-6^)/(6.5*0.8)*0.6*122 = **0.0734**

Pb excreted by chicks (g Pb bird y^-1^ in the colony) = (28.43* 240^1,06^ *7.65x10^-6^)/(6.5*0.8)*0.6*122*0.5/2 = **0.00349**

|  |  |  |
| --- | --- | --- |
| **Adult population** | **Chicks population^(1)^** | **Adults+chicks population^(2)^** |
|  |  |  |
| 8,200,000 | 2,050,000 | 10,250,000 |

(1): Population of chicks = Population adults (individuals not couples)*chicks by couple/2

(2): Population of adults and chicks (indiv.): Population (indiv) + Chicks****

Pb excreted by adults (g Pb y^-1^ in the colony) = 8,200,000*0.073350703 = 601,476 g Pb y^-1^

Pb excreted by chicks (g Pb y^-1^ in the colony) = 2,050,000*0.003488645 = 7,156 g Pb y^-1^

Pb excretated by adults + chicks (kg Pb y^-1^ in the colony) = (601,475.7672 + 7,151.722395)/1000 = **608.6 kg Pb y^-1^**

**References supplementary methods**

1. Paleczny, M.; Hammill, E.; Karpouzi, V.; Pauly, D. Population trend of the world’s monitored seabirds, 1950-2010. PLoS ONE 2015, 10, e0129342. DOI 10.1371/journal.pone.0129342
2. Riddick, S. N.; Dragosits, U.; Blackall, T. D.; Daunt, F.; Wanless, S.; Sutton, M. A. The global distribution of ammonia emissions from seabird colonies. Atmos. Environ. 2012, 55, 319–327. DOI 10.1016/j.atmosenv.2012.02.052
3. Otero, X. L.; De La Peña-Lastra, S.; Pérez-Alberti, A.; Ferreira, T. O.; Huerta-Díaz, M. A. Seabird colonies as important global drivers in the nitrogen and phosphorus cycles. Nat. Commun. 2018, 9, 246. DOI 10.1038/s41467-017-02446-8
4. Blackall, T. D.; Wilson, L. J.; Theobald, M. R.; Milford, C.; Nemitz, E.; Bull, J.; Bacon, P. J.; Hamer, K. C.; Wanless, S.; Sutton, M. A. Ammonia emissions from seabird colonies. Geophys. Res. Lett. 2007, 34, 5–17. DOI 10.1029/2006GL028928
5. Wilson, L. J.; Bacon, P. J.; Bull, J.; Dragosits, U.; Blackall, T. D.; Dunn, T. E.; Hamer, K. C.; Sutton, M. A.; Wanless, S. Modelling the spatial distribution of ammonia emissions from seabirds in the UK. Environ. Pollut. 2004, 131, 173–185. DOI 10.1016/j.envpol.2004.02.008
6. Furness, R. W. The occurrence of burrow-nesting among birds and its influence on soil fertility and stability. In Symposia of the Zoological Society of London; The Environmental Impact of Burrowing Animals and Animal Burrows; Meadows, P. S. and Meadows, A., Eds.; Zoological Society of London: UK, 1991; pp. 53–67.
7. Riddick, S. N.; Blackall, T. D.; Dragosits, U.; Daunt, F.; Newell, M.; Braban, C. F.; Tang, Y. S.; Schmale, J.; Hill, P. W.; Wanless, S.; Trathan, P.; Sutton, M. A. Measurement of ammonia emissions from temperate and subpolar seabird colonies. Atmospheric Environ. 2016, 134, 40–50. DOI 10.1016/j.atmosenv.2016.03.016

**Supplementary Table S1**. Size of the worldwide seabird population of breeding adult seabirds and chicks (PBASC) arranged by species, with the corresponding amounts of Cd, Hg and Pb excreted.

|  |  |  |  |  |  |  |  |
| --- | --- | --- | --- | --- | --- | --- | --- |
| **Species** | **Common name** | **Order*** | **PBASC**** | **Total Cd**  **excreted** | **Total Pb**  **excreted** | **Total Hg excreted** | **Reference** |
|  |  |  |  |  |  |  |  |
|  |  |  |  |  |  |  |  |
|  |  |  |  | **(kg y^-1^)***** | | |  |
|  |  |  |  |  |  |  |  |
|  |  |  |  |  |  |  |  |
| *Aethia cristatella* | Crested auklet | C | 10,250,000 | 595.54 | 608.63 | 21.81 | (1-4) |
| *Aethia psittacula* | Parakeet auklet | C | 1,500,000 | 43.91 | 44.87 | 1.61 | (1-4) |
| *Aethia pusilla* | Least auklet | C | 30,000,000 | 698.19 | 713.53 | 25.57 | (1-4) |
| *Aethia pygmaea* | Whiskered auklet | C | 125,000 | 3.72 | 3.80 | 0.14 | (1-4) |
| *Alca torda* | Razorbill | C | 32,760 | 4.72 | 4.82 | 0.17 | (2, 5) |
| *Alle alle* | Little auk | C | 32,500,000 | 1227.89 | 1254.86 | 44.96 | (2-4) |
| *Anous minutus* | Black noddy | C | 1,336,250 | 42.53 | 43.47 | 1.56 | (2, 4, 6) |
| *Anous stolidus* | Brown noddy | C | 800,000 | 37.83 | 38.66 | 1.39 | (2, 4, 6) |
| *Anous tenuirostris* | Lesser noddy | C | 1,536,000 | 41.57 | 42.48 | 1.52 | (2, 4, 6) |
| *Brachyramphus brevirostris* | Kittlitz's murrelet | C | 47,850 | 2.70 | 2.76 | 0.10 | (2, 4, 7) |
| *Brachyramphus marmoratus* | Marbled murrelet | C | 320,833 | 22.70 | 23.20 | 0.83 | (2, 4, 8) |
| *Brachyramphus perdix* | Long-billed murrelet | C | 412,083 | 26.00 | 26.58 | 0.95 | (2, 4, 9) |
| *Catharacta antarctica* | Southern skua | C | 43,200 | 13.19 | 13.48 | 0.48 | (2-4) |
| *Catharacta chilensis* | Chilean skua | C | 9,000 | 1.93 | 1.98 | 0.07 | (2, 4, 10) |
| *Catharacta lonnbergi* | Brown skua | C | 11,200 | 3.42 | 3.50 | 0.13 | (4, 11) |
| *Catharacta maccormicki* | South polar skua | C | 13,125 | 4.10 | 4.19 | 0.15 | (2, 4) |
| *Catharacta skua* | Great skua | C | 45,174 | 11.09 | 11.33 | 0.41 | (2, 4) |
| *Cepphus carbo* | Spectacled guillemot | C | 218,160 | 12.30 | 12.57 | 0.45 | (2-4) |
| *Cepphus columba* | Pigeon guillemot | C | 222,005 | 9.41 | 9.62 | 0.34 | (2-4) |
| *Cepphus grylle* | Black guillemot | C | 1,472,499 | 63.19 | 64.57 | 2.31 | (2-4) |
| *Cerorhinca monocerata* | Rhinoceros auklet | C | 1,885,000 | 98.30 | 100.46 | 3.60 | (2-4) |
| *Chionis albus* | Snowy sheathbill | C | 16,667 | 5.78 | 5.91 | 0.21 | (2, 4, 12) |
| *Chionis minor* | Black-faced sheathbill | C | 13,563 | 4.70 | 4.81 | 0.17 | (2, 4) |
| *Chlidonias albostriatus* | Black-fronted tern | C | 7,124 | 0.21 | 0.22 | 0.01 | (2, 4) |
| *Chlidonias hybrida* | Whiskered tern | C | 880,380 | 19.58 | 20.01 | 0.72 | (2, 4, 6) |
| *Chlidonias leucopterus* | White-winged tern | C | 3,472,610 | 77.25 | 78.95 | 2.83 | (2, 4, 6) |
| *Chlidonias niger* | Black tern | C | 1,238,663 | 20.25 | 20.70 | 0.74 | (2, 4, 6) |
| *Creagrus furcatus* | Swallow-tailed gull | C | 41,125 | 6.69 | 6.84 | 0.24 | (2, 4, 6) |
| *Fratercula arctica* | Atlantic puffin | C | 9,600,514 | 465.59 | 475.82 | 17.05 | (2, 4, 13) |
| *Fratercula cirrhata* | Tufted puffin | C | 4,462,500 | 297.01 | 303.54 | 10.88 | (2-4) |
| *Fratercula corniculata* | Horned puffin | C | 1,608,000 | 101.50 | 103.73 | 3.72 | (2-4) |
| *Gygis alba* | Common white tern | C | 790,400 | 24.62 | 25.16 | 0.90 | (2-4) |
| *Gygis microrhyncha* | Little white tern | C | 12,800 | 0.35 | 0.35 | 0.01 | (2, 4) |
| *Hydrocoloeus minutus* | Little gull | C | 330,300 | 8.27 | 8.45 | 0.30 | (2-4) |
| *Hydroprogne caspia* | Caspian tern | C | 232,155 | 25.30 | 25.85 | 0.93 | (2-4) |
| *Larosterna inca* | Inca tern | C | 180,000 | 8.02 | 8.19 | 0.29 | (2, 4) |
| *Larus argentatus* | Herring gull | C | 2,160,275 | 386.87 | 395.37 | 14.17 | (2-4) |
| *Larus atlanticus* | Olrog's gull | C | 18,415 | 3.17 | 3.24 | 0.12 | (2, 4, 14, 15) |
| *Larus atricilla* | Laughing gull | C | 1,287,825 | 86.61 | 88.51 | 3.17 | (2, 4, 6) |
| *Larus audouinii* | Audouin's gull | C | 63,536 | 9.42 | 9.63 | 0.35 | (2, 4) |
| *Larus belcheri* | Belcher's gull | C | 5,528 | 0.65 | 0.67 | 0.02 | (2, 4) |
| *Larus brunnicephalus* | Brown-headed gull | C | 217,500 | 15.80 | 16.15 | 0.58 | (2, 4, 6) |
|  |  |  |  |  |  |  |  |

**Supplementary Table S1**. Cont.

|  |  |  |  |  |  |  |  |
| --- | --- | --- | --- | --- | --- | --- | --- |
| **Species** | **Common name** | **Order*** | **PBASC**** | **Total Cd**  **excreted** | **Total Pb**  **excreted** | **Total Hg excreted** | **Reference** |
|  |  |  |  |  |  |  |  |
|  |  |  |  |  |  |  |  |
|  |  |  |  | **(kg y^-1^)***** | | |  |
|  |  |  |  |  |  |  |  |
|  |  |  |  |  |  |  |  |
| *Larus bulleri* | Black-billed gull | C | 148,800 | 10.04 | 10.26 | 0.37 | (2, 4) |
| *Larus cachinnans* | Yellow-legged gull | C | 513,333 | 101.97 | 104.21 | 3.73 | (2, 4, 6) |
| *Larus californicus* | California gull | C | 931,500 | 38.13 | 38.97 | 1.40 | (2, 4, 6) |
| *Larus canus* | Common gull | C | 2,928,570 | 271.13 | 277.09 | 9.93 | (2, 4, 6) |
| *Larus cirrocephalus* | Grey-headed gull | C | 1,011,375 | 73.48 | 75.10 | 2.69 | (2, 4, 6) |
| *Larus crassirostris* | Black-tailed gull | C | 1,540,000 | 175.38 | 179.24 | 6.42 | (2, 4, 6) |
| *Larus delawarensis* | Ring-billed gull | C | 3,825,000 | 345.48 | 353.07 | 12.65 | (2, 4, 6) |
| *Larus dominicanus* | Kelp gull | C | 3,946,300 | 685.01 | 700.06 | 25.08 | (2, 4) |
| *Larus fuliginosus* | Lava gull | C | 1,225 | 0.07 | 0.07 | 0.00 | (2, 4, 6) |
| *Larus fuscus* | Lesser black-backed gull | C | 1,315,897 | 221.39 | 226.26 | 8.11 | (2, 4, 6) |
| *Larus genei* | Slender-billed gull | C | 346,725 | 22.00 | 22.49 | 0.81 | (2, 4, 6) |
| *Larus glaucescens* | Glaucous-winged gull | C | 883,500 | 217.81 | 222.60 | 7.98 | (2, 4, 6) |
| *Larus glaucoides* | Iceland gull | C | 306,358 | 43.30 | 44.25 | 1.59 | (2, 4, 6) |
| *Larus hartlaubii* | King gull | C | 45,000 | 3.10 | 3.17 | 0.11 | (2, 4, 6) |
| *Larus heermanni* | Heermann's gull | C | 405,000 | 45.41 | 46.41 | 1.66 | (2, 4, 16) |
| *Larus hemprichii* | Sooty gull | C | 337,500 | 26.23 | 26.81 | 0.96 | (2, 4, 6) |
| *Larus hyperboreus* | Glaucous gull | C | 954,750 | 198.79 | 203.15 | 7.28 | (2, 4, 6) |
| *Larus ichthyaetus* | Pallas's gull | C | 697,638 | 158.93 | 162.43 | 5.82 | (2, 4, 6) |
| *Larus leucophthalmus* | White-eyed gull | C | 60,750 | 4.72 | 4.83 | 0.17 | (2, 4, 6) |
| *Larus livens* | Yellow-footed gull | C | 75,000 | 15.25 | 15.59 | 0.56 | (2, 4, 6) |
| *Larus maculipennis* | Brown-hooded gull | C | 834,000 | 56.79 | 58.04 | 2.08 | (2, 4, 6) |
| *Larus marinus* | Great black-backed gull | C | 413,718 | 101.80 | 104.04 | 3.73 | (2, 4, 6) |
| *Larus melanocephalus* | Mediterranean gull | C | 2,145,999 | 122.94 | 125.64 | 4.50 | (2, 4, 6) |
| *Larus modestus* | Gray gull | C | 47,500 | 3.13 | 3.20 | 0.11 | (2, 4, 6) |
| *Larus novaehollandiae* | Silver gull | C | 770,000 | 63.16 | 64.55 | 2.31 | (2, 4, 6) |
| *Larus occidentalis* | Western gull | C | 152,100 | 34.83 | 35.59 | 1.28 | (2, 4, 6) |
| *Larus pacificus* | Pacific gull | C | 16,425 | 2.92 | 2.98 | 0.11 | (2, 4, 6) |
| *Larus philadelphia* | Bonaparte's gull | C | 585,000 | 29.33 | 29.98 | 1.07 | (2, 4, 6) |
| *Larus pipixcan* | Franklin's gull | C | 1,245,000 | 80.53 | 82.30 | 2.95 | (2, 4, 6) |
| *Larus relictus* | Relict gull | C | 21,750 | 1.34 | 1.37 | 0.05 | (2, 4) |
| *Larus ridibundus* | Black-headed gull | C | 6,884,250 | 436.53 | 446.12 | 15.98 | (2, 4, 6) |
| *Larus schistisagus* | Slaty-backed gull | C | 515,063 | 118.90 | 121.51 | 4.35 | (2, 4, 6) |
| *Larus scopulinus* | Red-billed gull | C | 852,500 | 57.52 | 58.78 | 2.11 | (4, 6) |
| *Larus scoresbii* | Dolphin gull | C | 18,376 | 1.94 | 1.99 | 0.07 | (2, 4, 6) |
| *Larus serranus* | Andean gull | C | 145,000 | 12.89 | 13.18 | 0.47 | (2, 4, 6) |
| *Larus thayeri* | Thayer's gull | C | 26,250 | 5.05 | 5.16 | 0.18 | (2, 4, 6) |
| *Onychoprion aleuticus* | Aleutian tern | C | 44,175 | 1.25 | 1.28 | 0.05 | (2, 4, 6) |
| *Onychoprion anaethetus* | Bridled tern | C | 993,124 | 32.25 | 32.96 | 1.18 | (2, 4, 6) |
| *Onychoprion fuscatus* | Sooty tern | C | 18,006,250 | 819.57 | 837.58 | 30.01 | (2, 4, 6) |
| *Pagophila eburnea* | Ivory gull | C | 67,500 | 18.46 | 18.87 | 0.68 | (2, 4, 6) |
| *Phaetusa simplex* | Large-billed tern | C | 69,333 | 5.89 | 6.02 | 0.22 | (2, 4, 6) |
|  |  |  |  |  |  |  |  |

**Supplementary Table S1**. Cont.

|  |  |  |  |  |  |  |  |
| --- | --- | --- | --- | --- | --- | --- | --- |
| **Species** | **Common name** | **Order*** | **PBASC**** | **Total Cd**  **excreted** | **Total Pb**  **excreted** | **Total Hg excreted** | **Reference** |
|  |  |  |  |  |  |  |  |
|  |  |  |  |  |  |  |  |
|  |  |  |  | **(kg y^-1^)***** | | |  |
|  |  |  |  |  |  |  |  |
|  |  |  |  |  |  |  |  |
| *Procelsterna cerulea* | Blue noddy | C | 61,556 | 0.84 | 0.85 | 0.03 | (2, 4) |
| *Ptychoramphus aleuticus* | Cassin's auklet | C | 4,874,999 | 123.68 | 126.39 | 4.53 | (2, 4, 17) |
| *Rhodostethia rosea* | Ross's gull | C | 62,813 | 3.12 | 3.19 | 0.11 | (2, 4, 6) |
| *Rissa brevirostris* | Red-legged kittiwake | C | 3,374,999 | 443.06 | 452.80 | 16.22 | (2, 4) |
| *Rissa tridactyla* | Black-legged kittiwake | C | 14,109,195 | 1270.91 | 1298.84 | 46.54 | (2, 4, 6) |
| *Saundersilarus saundersi* | Saunders's gull | C | 20,880 | 1.32 | 1.35 | 0.05 | (1, 4) |
| *Stercorarius longicaudus* | Long-tailed skua | C | 5,625,000 | 556.49 | 568.72 | 20.38 | (2, 4) |
| *Stercorarius parasiticus* | Parasitic skua | C | 6,225,000 | 694.37 | 709.63 | 25.43 | (2-4) |
| *Stercorarius pomarinus* | Pomarine skua | C | 556,000 | 136.44 | 139.44 | 5.00 | (2-4) |
| *Sterna acuticauda* | Black-bellied tern | C | 17,301 | 0.38 | 0.39 | 0.01 | (2, 4) |
| *Sterna aurantia* | River tern | C | 59,044 | 3.46 | 3.53 | 0.13 | (2, 4, 18) |
| *Sterna dougallii* | Roseate tern | C | 245,522 | 4.29 | 4.39 | 0.16 | (2, 4) |
| *Sterna forsteri* | Forster's tern | C | 65,858 | 2.33 | 2.38 | 0.09 | (2, 4) |
| *Sterna hirundinacea* | South American tern | C | 602,188 | 29.01 | 29.65 | 1.06 | (2, 4, 6) |
| *Sterna hirundo* | Common tern | C | 2,830,750 | 73.18 | 74.79 | 2.68 | (2, 4, 6) |
| *Sterna nilotica* | Gull-billed tern | C | 332,253 | 10.73 | 10.97 | 0.39 | (2, 4, 6) |
| *Sterna paradisaea* | Arctic tern | C | 2,800,000 | 70.06 | 71.60 | 2.57 | (2, 4, 6) |
| *Sterna repressa* | White-cheeked tern | C | 876,000 | 24.43 | 24.97 | 0.89 | (2, 4, 6) |
| *Sterna striata* | White-fronted tern | C | 37,800 | 1.56 | 1.60 | 0.06 | (2, 4, 6) |
| *Sterna sumatrana* | Black-naped tern | C | 101,603 | 3.17 | 3.24 | 0.12 | (2, 4) |
| *Sterna superciliaris* | Yellow-billed tern | C | 55,417 | 0.83 | 0.85 | 0.03 | (2, 4, 6) |
| *Sterna trudeaui* | Snowy-crowned tern | C | 4,791 | 0.17 | 0.18 | 0.01 | (2-4) |
| *Sterna virgata* | Kerguelen tern | C | 3,696 | 0.14 | 0.14 | 0.01 | (2, 4) |
| *Sterna vittata* | Antarctic tern | C | 118,379 | 4.16 | 4.25 | 0.15 | (2, 4, 6) |
| *Sternula albifrons* | Little tern | C | 391,950 | 3.66 | 3.74 | 0.13 | (2, 4, 6) |
| *Sternula antillarum* | Least tern | C | 70,578 | 0.91 | 0.93 | 0.03 | (2, 4, 6) |
| *Sternula balaenarum* | Damara tern | C | 4,718 | 0.06 | 0.06 | 0.00 | (2-4) |
| *Sternula lorata* | Peruvian tern | C | 1,380 | 0.02 | 0.02 | 0.00 | (2, 4) |
| *Sternula nereis* | Fairy tern | C | 6,874 | 0.16 | 0.17 | 0.01 | (2, 4, 6) |
| *Sternula saundersi* | Saunders's tern | C | 38,000 | 0.46 | 0.47 | 0.02 | (2, 4, 6) |
| *Synthliboramphus antiquus* | Ancient murrelet | C | 1,778,850 | 21.69 | 22.16 | 0.79 | (2-4) |
| *Synthliboramphus craveri* | Craveri's murrelet | C | 12,000 | 0.19 | 0.19 | 0.01 | (*2, 4, 19*) |
| *Synthliboramphus hypoleucus* | Xantus's murrelet | C | 6,800 | 0.12 | 0.13 | 0.00 | (2*, 4, 20*) |
| *Synthliboramphus wumizusume* | Japanese murrelet | C | 7,374 | 0.33 | 0.34 | 0.01 | (2, 4) |
| *Thalasseus bengalensis* | Lesser crested tern | C | 256,667 | 11.88 | 12.14 | 0.43 | (2, 4, 6) |
| *Thalasseus bergii* | Greater crested tern | C | 492,031 | 38.06 | 38.90 | 1.39 | (2, 4) |
| *Thalasseus bernsteini* | Chinese crested tern | C | 46 | 0.00 | 0.00 | 0.00 | (2, 4) |
| *Thalasseus elegans* | Elegant tern | C | 90,240 | 4.98 | 5.09 | 0.18 | (2, 4, 16) |
| *Thalasseus maximus* | Royal tern | C | 479,872 | 43.13 | 44.08 | 1.58 | (2, 4, 6) |
| *Thalasseus sandvicensis* | Sandwich tern | C | 569,718 | 26.68 | 27.27 | 0.98 | (2, 4, 6) |
| *Uria aalge* | Common guillemot | C | 24,390,000 | 4486.47 | 4585.04 | 164.28 | (2-4) |
|  |  |  |  |  |  |  |  |

**Supplementary Table S1**. Cont.

|  |  |  |  |  |  |  |  |
| --- | --- | --- | --- | --- | --- | --- | --- |
| **Species** | **Common name** | **Order*** | **PBASC**** | **Total Cd**  **excreted** | **Total Pb**  **excreted** | **Total Hg excreted** | **Reference** |
|  |  |  |  |  |  |  |  |
|  |  |  |  |  |  |  |  |
|  |  |  |  | **(kg y^-1^)***** | | |  |
|  |  |  |  |  |  |  |  |
|  |  |  |  |  |  |  |  |
| *Uria lomvia* | Thick-billed murre | C | 30,800,000 | 4383.70 | 4480.02 | 160.52 | (2-4) |
| *Xema sabini* | Sabines gull | C | 415,785 | 24.32 | 24.85 | 0.89 | (2, 4, 6) |
|  |  |  |  |  |  |  |  |
| **Total for this order:** |  | **C** | **266,421,480** | **21,705** | **22,182** | **794.79** |  |
|  |  |  |  |  |  |  |  |
| *Fregata andrewsi* | Christmas Island frigatebird | Pe | 3,960 | 0.64 | 0.17 | 4.43 | (*2, 4, 21*) |
| *Fregata aquila* | Ascension frigatebird | Pe | 20,615 | 3.68 | 1.00 | 25.57 | (2-4) |
| *Fregata ariel* | Lesser frigatebird | Pe | 220,000 | 22.02 | 5.97 | 153.06 | (2-4) |
| *Fregata magnificens* | Magnificent frigatebird | Pe | 44,000 | 7.75 | 2.10 | 53.86 | (2-4) |
| *Fregata minor* | Great Frigatebird | Pe | 881,250 | 116.94 | 31.72 | 812.92 | (2-4) |
| *Microcarbo africanus* | Reed Cormorant | Pe | 537,817 | 32.40 | 8.79 | 225.24 | (2, 4, 6) |
| *Microcarbo coronatus* | Crowned Cormorant | Pe | 10,385 | 0.63 | 0.17 | 4.37 | (2, 4) |
| *Microcarbo melanoleucos* | Little pied cormorant | Pe | 150,100 | 9.65 | 2.62 | 67.09 | (2, 4, 6) |
| *Microcarbo niger* | Little cormorant | Pe | 468,750 | 14.81 | 4.02 | 102.95 | (2, 4, 6) |
| *Microcarbo pygmaeus* | Pygmy cormorant | Pe | 161,135 | 4.32 | 1.17 | 30.00 | (2, 4, 6) |
| *Morus bassanus* | Northern gannet | Pe | 2,252,250 | 576.00 | 156.25 | 4004.11 | (2-4) |
| *Morus capensis* | Cape gannet | Pe | 339,480 | 80.82 | 21.92 | 561.84 | (2-4) |
| *Morus serrator* | Australasian gannet | Pe | 281,585 | 46.27 | 12.55 | 321.68 | (*2, 4, 22*) |
| *Papasula abbotti* | Abbott's booby | Pe | 6,600 | 1.37 | 0.37 | 9.49 | (2-4) |
| *Pelecanus occidentalis* | Brown pelican | Pe | 396,976 | 117.44 | 31.86 | 816.37 | (2-4) |
| *Phaethon aethereus* | Red-billed tropicbird | Pe | 10,025 | 0.53 | 0.14 | 3.67 | (2-4) |
| *Phaethon lepturus* | White-tailed tropicbird | Pe | 60,750 | 2.99 | 0.81 | 20.75 | (2-4) |
| *Phaethon rubricauda* | Red-tailed tropicbird | Pe | 39,680 | 2.56 | 0.70 | 17.82 | (2-4) |
| *Phalacrocorax aristotelis* | European shag | Pe | 262,942 | 37.41 | 10.15 | 260.08 | (2, 4, 6) |
| *Phalacrocorax atriceps* | Imperial shag | Pe | 1,435,081 | 215.66 | 58.50 | 1499.17 | (2, 4, 6) |
| *Phalacrocorax auritus* | Double-crested cormorant | Pe | 1,291,082 | 200.63 | 54.42 | 1394.71 | (2, 4, 6) |
| *Phalacrocorax bougainvilliorum* | Guanay Cormorant | Pe | 8,249,999 | 1058.50 | 287.13 | 7358.25 | (2-4) |
| *Phalacrocorax brasilianus* | Neotropic cormorant | Pe | 3,650,000 | 206.40 | 55.99 | 1434.77 | (2-4) |
| *Phalacrocorax campbelli* | Campbell Island shag | Pe | 9,600 | 1.30 | 0.35 | 9.06 | (2, 4, 6) |
| *Phalacrocorax capensis* | Cape cormorant | Pe | 391,950 | 36.48 | 9.90 | 253.61 | (2, 4) |
| *Phalacrocorax capillatus* | Japanese cormorant | Pe | 92,125 | 15.51 | 4.21 | 107.80 | (2, 4, 6) |
| *Phalacrocorax carbo* | Great cormorant | Pe | 2,438,800 | 270.39 | 73.35 | 1879.64 | (2, 4, 6) |
| *Phalacrocorax carunculatus* | King shag | Pe | 781 | 0.15 | 0.04 | 1.06 | (2, 4) |
| *Phalacrocorax chalconotus* | Stewart Island shag | Pe | 2,160 | 0.50 | 0.14 | 3.49 | (2, 4) |
| *Phalacrocorax colensoi* | Auckland shag | Pe | 3,600 | 0.49 | 0.13 | 3.40 | (2, 4) |
| *Phalacrocorax featherstoni* | Pitt cormorant | Pe | 1,042 | 0.18 | 0.05 | 1.23 | (2-4) |
| *Phalacrocorax fuscescens* | Black-faced shag | Pe | 11,167 | 1.08 | 0.29 | 7.52 | (2-4) |
| *Phalacrocorax fuscicollis* | Indian cormorant | Pe | 67,500 | 2.13 | 0.58 | 14.83 | (2, 4, 6) |
| *Phalacrocorax gaimardi* | Red-legged cormorant | Pe | 58,960 | 5.10 | 1.38 | 35.47 | (2, 4) |
| *Phalacrocorax harrisi* | Flightless cormorant | Pe | 3,605 | 1.25 | 0.34 | 8.68 | (2, 4) |
| *Phalacrocorax magellanicus* | Rock shag | Pe | 205,333 | 27.75 | 7.53 | 192.89 | (2, 4, 6) |
|  |  |  |  |  |  |  |  |

**Supplementary Table S1**. Cont.

|  |  |  |  |  |  |  |  |
| --- | --- | --- | --- | --- | --- | --- | --- |
| **Species** | **Common name** | **Order*** | **PBASC**** | **Total Cd**  **excreted** | **Total Pb**  **excreted** | **Total Hg excreted** | **Reference** |
|  |  |  |  |  |  |  |  |
|  |  |  |  |  |  |  |  |
|  |  |  |  | **(kg y^-1^)***** | | |  |
|  |  |  |  |  |  |  |  |
|  |  |  |  |  |  |  |  |
| *Phalacrocorax neglectus* | Bank cormorant | Pe | 8,750 | 1.09 | 0.30 | 7.59 | (2, 4) |
| *Phalacrocorax nigrogularis* | Socotra cormorant | Pe | 368,500 | 56.78 | 15.40 | 394.70 | (*2, 4, 23*) |
| *Phalacrocorax onslowi* | Chatham Island shag | Pe | 1,065 | 0.18 | 0.05 | 1.24 | (2, 4) |
| *Phalacrocorax pelagicus* | Pelagic Cormorant | Pe | 24,500 | 3.00 | 0.81 | 20.85 | (2, 4, 6) |
| *Phalacrocorax penicillatus* | Brandt's cormorant | Pe | 130,000 | 17.55 | 4.76 | 122.02 | (2, 4, 6) |
| *Phalacrocorax punctatus* | Spotted shag | Pe | 187,313 | 19.53 | 5.30 | 135.74 | (2, 4, 6) |
| *Phalacrocorax ranfurlyi* | Bounty Islands shag | Pe | 1,575 | 0.24 | 0.06 | 1.66 | (2, 4) |
| *Phalacrocorax sulcirostris* | Little black shag | Pe | 678,400 | 19.58 | 5.31 | 136.10 | (2, 4, 6) |
| *Phalacrocorax urile* | Red-faced cormorant | Pe | 325,000 | 45.02 | 12.21 | 312.99 | (2, 4, 6) |
| *Phalacrocorax varius* | Greater pied cormorant | Pe | 89,067 | 12.67 | 3.44 | 88.09 | (2, 4, 6) |
| *Phalacrocorax verrucosus* | Kerguelen shag | Pe | 7,367 | 1.11 | 0.30 | 7.70 | (2-4) |
| *Sula dactylatra* | Masked booby | Pe | 112,950 | 25.20 | 6.84 | 175.20 | (2-4) |
| *Sula granti* | Nazca booby | Pe | 37,500 | 3.59 | 0.97 | 24.95 | (2-4) |
| *Sula leucogaster* | Brown booby | Pe | 280,000 | 32.87 | 8.92 | 228.49 | (2-4) |
| *Sula nebouxii* | Blue-footed booby | Pe | 98,000 | 17.88 | 4.85 | 124.28 | (*2, 4, 24*) |
| *Sula sula* | Red-footed booby | Pe | 1,200,000 | 144.31 | 39.15 | 1003.16 | (2-4) |
| *Sula variegata* | Peruvian booby | Pe | 1,421,000 | 192.35 | 52.18 | 1337.11 | (2-4) |
|  |  |  |  |  |  |  |  |
| **Total for this order:** |  | **Pe** | **29,032,072** | **3,715** | **1,008** | **25,823** |  |
|  |  |  |  |  |  |  |  |
| *Aphrodroma brevirostris* | Kerguelen petrel | Pr | 1,500,000 | 5.48 | 1.02 | 5.29 | (*2, 4, 25*) |
| *Ardenna bulleri* | Buller's shearwater | Pr | 2,010,000 | 10.65 | 1.99 | 10.29 | (*2, 4, 26*) |
| *Ardenna carneipes* | Flesh-footed shearwater | Pr | 802,750 | 12.04 | 2.24 | 11.63 | (2, 4, 25) |
| *Ardenna creatopus* | Pink-footed shearwater | Pr | 73,933 | 0.87 | 0.16 | 0.84 | (2, 4) |
| *Ardenna gravis* | Great shearwater | Pr | 19,950,000 | 423.16 | 78.91 | 408.87 | (2, 4, 25) |
| *Ardenna grisea* | Sooty shearwater | Pr | 25,500,000 | 221.57 | 41.32 | 214.09 | (2, 4, 25) |
| *Ardenna pacifica* | Wedge-tailed Shearwater | Pr | 6,370,000 | 32.61 | 6.08 | 31.51 | (2, 4, 25) |
| *Ardenna tenuirostris* | Short-tailed Shearwater | Pr | 29,900,000 | 1638.61 | 305.58 | 1583.27 | (2, 4, 25) |
| *Bulweria bulwerii* | Bulwer’s petrel | Pr | 613,050 | 7.08 | 1.32 | 6.84 | (2, 4, 25) |
| *Bulweria fallax* | Jouanin's petrel | Pr | 5,206 | 0.08 | 0.01 | 0.07 | (2, 4, 25) |
| *Calonectris diomedea* | Cory's shearwater | Pr | 456,875 | 21.04 | 3.92 | 20.33 | (2, 4) |
| *Calonectris edwardsii* | Cape Verde shearwater | Pr | 17,250 | 0.79 | 0.15 | 0.77 | (2, 4) |
| *Calonectris leucomelas* | Streaked shearwater | Pr | 2,412,000 | 64.52 | 12.03 | 62.34 | (2, 4, 25) |
| *Daption capense* | Cape petrel | Pr | 2,500,000 | 64.12 | 11.96 | 61.96 | (2, 4, 25) |
| *Daption capense australe* | Snares Cape pigeon | Pr | 6,250 | 0.16 | 0.03 | 0.15 | (3, 4) |
| *Diomedea amsterdamensis* | Amsterdam albatross | Pr | 135 | 0.06 | 0.01 | 0.06 | (2-4) |
| *Diomedea antipodensis* | Antipodean albatross | Pr | 55,000 | 43.03 | 8.02 | 41.58 | (2-4) |
| *Diomedea dabbenena* | Tristan albatross | Pr | 4,777 | 1.97 | 0.37 | 1.90 | (2-4) |
| *Diomedea epomophora* | Southern royal albatross | Pr | 35,632 | 17.12 | 3.19 | 16.54 | (2-4) |
| *Diomedea exulans* | Wandering albatross | Pr | 23,417 | 11.23 | 2.09 | 10.85 | (2-4) |
| *Diomedea sanfordi* | Northern royal albatross | Pr | 19,635 | 13.13 | 2.45 | 12.68 | (2-4) |
| *Fregetta grallaria* | White-bellied storm-petrel | Pr | 375,000 | 0.36 | 0.07 | 0.35 | (2-4) |
|  |  |  |  |  |  |  |  |

**Supplementary Table S1**. Cont.

|  |  |  |  |  |  |  |  |
| --- | --- | --- | --- | --- | --- | --- | --- |
| **Species** | **Common name** | **Order*** | **PBASC**** | **Total Cd**  **excreted** | **Total Pb**  **excreted** | **Total Hg excreted** | **Reference** |
|  |  |  |  |  |  |  |  |
|  |  |  |  |  |  |  |  |
|  |  |  |  | **(kg y^-1^)***** | | |  |
|  |  |  |  |  |  |  |  |
|  |  |  |  |  |  |  |  |
| *Fregetta maoriana* | New Zealand storm-petrel | Pr | 31 | --- | --- | --- | (2, 4, 25) |
| *Fregetta tropica* | Black-bellied Storm-petrel | Pr | 625,000 | 0.61 | 0.11 | 0.59 | (2, 4, 25) |
| *Fulmarus glacialis* | Northern fulmar | Pr | 25,700,000 | 1,791.46 | 334.08 | 1,730.95 | (2, 4) |
| *Fulmarus glacialoides* | Southern fulmar | Pr | 5,140,000 | 216.85 | 40.44 | 209.53 | (2, 4, 25) |
| *Garrodia nereis* | Grey-backed storm-petrel | Pr | 250,000 | 0.13 | 0.02 | 0.12 | (2, 4, 25) |
| *Halobaena caerulea* | Blue petrel | Pr | 3,750,000 | 26.85 | 5.01 | 25.94 | (2, 4, 25) |
| *Hydrobates castro* | Madeiran storm-petrel | Pr | 180,000 | 0.23 | 0.04 | 0.22 | (2, 4, 25) |
| *Hydrobates furcatus* | Fork tailed storm-petrel | Pr | 8,100,000 | 6.62 | 1.23 | 6.40 | (2, 4, 25) |
| *Hydrobates homochroa* | Ashy storm-petrel | Pr | 6,630 | 0.01 | - | 0.01 | (2, 4, 25) |
| *Hydrobates hornbyi* | Hornby's storm-petrel | Pr | 37,160 | 0.03 | 0.01 | 0.03 | (2, 4, 25) |
| *Hydrobates leucorhous* | Leach's storm-petrel | Pr | 9,375,000 | 9.87 | 1.84 | 9.54 | (2, 4, 25) |
| *Hydrobates markhami* | Markham's storm-petrel | Pr | 61,250 | 0.10 | 0.02 | 0.10 | (2, 4, 25) |
| *Hydrobates matsudairae* | Matsudaira's storm-petrel | Pr | 24,500 | 0.02 | - | 0.02 | (2, 4, 25) |
| *Hydrobates melania* | Black storm-petrel | Pr | 650,000 | 0.66 | 0.12 | 0.64 | (2, 4, 25) |
| *Hydrobates microsoma* | Least storm petrel | Pr | 706,250 | 0.35 | 0.06 | 0.34 | (2-4) |
| *Hydrobates monorhis* | Swinhoe's storm-petrel | Pr | 338,000 | 0.36 | 0.07 | 0.35 | (2, 4, 25) |
| *Hydrobates monteiroi* | Monteiro's storm-petrel | Pr | 749 | --- | --- | --- | (2, 4, 25) |
| *Hydrobates pelagicus* | European storm-petrel | Pr | 593,750 | 0.36 | 0.07 | 0.35 | (2, 4, 25) |
| *Hydrobates tethys* | Wedge-rumped storm-petrel | Pr | 600,000 | 0.34 | 0.06 | 0.33 | (2, 4, 25) |
| *Macronectes giganteus* | Southern giant-petrel | Pr | 118,800 | 29.72 | 5.54 | 28.72 | (2, 4) |
| *Macronectes halli* | Northern giant-petrel | Pr | 34,220 | 6.01 | 1.12 | 5.81 | (2, 4) |
| *Nesofregetta fuliginosa* | White-throated storm-petrel | Pr | 781 | --- | --- | --- | (2, 4, 25) |
| *Oceanites gracilis* | White-vented storm-petrel | Pr | 36,750 | 0.04 | 0.01 | 0.04 | (2, 4, 25) |
| *Oceanites oceanicus* | Wilson's Storm-petrel | Pr | 25,200,000 | 28.52 | 5.32 | 27.56 | (2, 4, 25) |
| *Oceanodroma tristrami* | Tristram’s Storm-petrel | Pr | 24,500 | 0.02 | --- | 0.02 | (2, 4, 25) |
| *Pachyptila belcheri* | Slender-billed prion | Pr | 8,330,000 | 59.93 | 11.18 | 57.91 | (2, 4, 25) |
| *Pachyptila crassirostris* | Fulmar prion | Pr | 281,250 | 1.74 | 0.33 | 1.69 | (2, 4, 25) |
| *Pachyptila desolata* | Antarctic prion | Pr | 62,500,000 | 386.40 | 72.06 | 373.35 | (2, 4, 25) |
| *Pachyptila salvini* | Medium-billed prion | Pr | 15,000,000 | 103.69 | 19.34 | 100.19 | (2, 4, 25) |
| *Pachyptila turtur* | Fairy prion | Pr | 6,250,000 | 38.77 | 7.23 | 37.46 | (2, 4, 25) |
| *Pachyptila vittata* | Broad-billed prion | Pr | 18,750,000 | 155.67 | 29.03 | 150.41 | (2, 4, 25) |
| *Pagodroma nivea* | Snow petrel | Pr | 5,000,000 | 77.65 | 14.48 | 75.03 | (2, 4, 25) |
| *Pelagodroma marina* | White-faced storm-petrel | Pr | 5,200,000 | 5.08 | 0.95 | 4.91 | (2, 4, 25) |
| *Pelecanoides georgicus* | South Georgia diving-petrel | Pr | 20,250,000 | 111.03 | 20.71 | 107.28 | (2, 4, 25) |
| *Pelecanoides urinatrix* | Common diving-petrel | Pr | 22,400,000 | 121.66 | 22.69 | 117.55 | (2, 4, 25) |
| *Phoebastria albatrus* | Short-tailed albatross | Pr | 2,176 | 0.74 | 0.14 | 0.71 | (2-4) |
| *Phoebastria immutabilis* | Laysan albatross | Pr | 1,984,000 | 628.79 | 117.26 | 607.56 | (2-4) |
| *Phoebastria irrorata* | Waved albatross | Pr | 39,038 | 14.77 | 2.75 | 14.27 | (2-4) |
| *Phoebastria nigripes* | Black-footed albatross | Pr | 173,510 | 54.17 | 10.10 | 52.34 | (2-4) |
| *Phoebetria fusca* | Sooty albatross | Pr | 27,689 | 3.70 | 0.69 | 3.58 | (2-4) |
| *Phoebetria palpebrata* | Light-mantled albatross | Pr | 76,270 | 23.56 | 4.39 | 22.76 | (2-4) |
| *Procellaria aequinoctialis* | White-chinned petrel | Pr | 2,988,000 | 110.21 | 20.55 | 106.49 | (2, 4, 25) |
| *Procellaria cinerea* | Grey petrel | Pr | 540,000 | 15.66 | 2.92 | 15.13 | (2, 4, 25) |
|  |  |  |  |  |  |  |  |

**Supplementary Table S1**. Cont.

|  |  |  |  |  |  |  |  |
| --- | --- | --- | --- | --- | --- | --- | --- |
| **Species** | **Common name** | **Order*** | **PBASC**** | **Total Cd**  **excreted** | **Total Pb**  **excreted** | **Total Hg excreted** | **Reference** |
|  |  |  |  |  |  |  |  |
|  |  |  |  |  |  |  |  |
|  |  |  |  | **(kg y^-1^)***** | | |  |
|  |  |  |  |  |  |  |  |
|  |  |  |  |  |  |  |  |
| *Procellaria conspicillata* | Spectacled petrel | Pr | 24,500 | 0.32 | 0.06 | 0.31 | (2, 4) |
| *Procellaria parkinsoni* | Parkinson's petrel | Pr | 6,738 | 0.15 | 0.03 | 0.15 | (2, 4) |
| *Procellaria westlandica* | Westland petrel | Pr | 13,108 | 0.50 | 0.09 | 0.48 | (2, 4) |
| *Pseudobulweria aterrima* | Mascarene petrel | Pr | 184 | --- | --- | --- | (2, 4) |
| *Pseudobulweria becki* | Beck's petrel | Pr | 183 | --- | --- | --- | (2, 4) |
| *Pseudobulweria macgillivrayi* | Fiji petrel | Pr | 31 | --- | --- | --- | (2, 4) |
| *Pseudobulweria rostrata* | Tahiti petrel | Pr | 18,749 | 0.13 | 0.02 | 0.13 | (2, 4) |
| *Pterodrom leucoptera* | Gould's petrel | Pr | 9,800 | 0.08 | 0.02 | 0.08 | (2, 4, 25) |
| *Pterodroma alba* | Phoenix petrel | Pr | 37,500 | 0.26 | 0.05 | 0.25 | (2, 4) |
| *Pterodroma arminjoniana* | Trindade petrel | Pr | 2,769 | 0.09 | 0.02 | 0.09 | (*2, 4, 27*) |
| *Pterodroma atrata* | Henderson petrel | Pr | 44,000 | 1.59 | 0.30 | 1.53 | (*2, 4, 25, 28*) |
| *Pterodroma axillaris* | Chatham petrel | Pr | 1,348 | 0.02 | --- | 0.02 | (2, 4, 25) |
| *Pterodroma baraui* | Barau's petrel | Pr | 38,500 | 0.99 | 0.18 | 0.96 | (2, 4, 25) |
| *Pterodroma brevipes* | Collared petrel | Pr | 4,054 | 0.04 | 0.01 | 0.04 | (2, 4, 25) |
| *Pterodroma cahow* | Bermuda petrel | Pr | 160 | 0.01 | --- | --- | (2, 4, 25) |
| *Pterodroma cookie* | Cook's petrel | Pr | 804,000 | 7.07 | 1.32 | 6.83 | (2, 4, 25) |
| *Pterodroma defilippiana* | De Filippi's petrel | Pr | 6,804 | 0.06 | 0.01 | 0.06 | (2, 4, 25) |
| *Pterodroma externa* | Juan Fernandez petrel | Pr | 2,261,250 | 58.98 | 11.00 | 56.98 | (2, 4, 25) |
| *Pterodroma feae* | Fea's petrel | Pr | 1,650 | 0.02 | --- | 0.02 | (2, 4, 25) |
| *Pterodroma hasitata* | Black-capped petrel | Pr | 1,838 | 0.02 | --- | 0.02 | (2, 4, 25) |
| *Pterodroma heraldica* | Herald petrel | Pr | 172,500 | 2.22 | 0.41 | 2.14 | (2, 4, 25) |
| *Pterodroma hypoleuca* | Bonin petrel | Pr | 1,125,000 | 8.94 | 1.67 | 8.64 | (2, 4, 25) |
| *Pterodroma incerta* | Atlantic petrel | Pr | 1,980,000 | 40.02 | 7.46 | 38.67 | (2, 4) |
| *Pterodroma inexpectata* | Mottled petrel | Pr | 424,000 | 6.76 | 1.26 | 6.53 | (*2, 4, 29*) |
| *Pterodroma lessonii* | White-headed petrel | Pr | 645,000 | 29.82 | 5.56 | 28.81 | (2, 4, 25) |
| *Pterodroma longirostris* | Stejneger's petrel | Pr | 500,000 | 5.06 | 0.94 | 4.89 | (2, 4) |
| *Pterodroma macroptera* | Great-winged petrel | Pr | 1,650,000 | 32.40 | 6.04 | 31.30 | (2, 4, 25) |
| *Pterodroma magenta* | Magenta petrel | Pr | 110 | --- | --- | --- | (2, 4) |
| *Pterodroma mollis* | Soft-plumaged petrel | Pr | 6,000,000 | 75.61 | 14.10 | 73.05 | (2, 4, 25) |
| *Pterodroma neglecta* | Kermadec petrel | Pr | 183,750 | 7.52 | 1.40 | 7.26 | (2, 4, 25) |
| *Pterodroma nigripennis* | Black-winged petrel | Pr | 12,375,000 | 180.51 | 33.66 | 174.41 | (2, 4, 25) |
| *Pterodroma phaeopygia* | Galapagos petrel | Pr | 10,640 | 0.17 | 0.03 | 0.16 | (2, 4) |
| *Pterodroma pycrofti* | Pycroft's petrel | Pr | 21,250 | 0.18 | 0.03 | 0.17 | (2, 4) |
| *Pterodroma sandwichensis* | Hawaiian petrel | Pr | 10,625 | 0.43 | 0.08 | 0.42 | (2, 4, 25) |
| *Pterodroma solandri* | Providence petrel | Pr | 117,500 | 1.90 | 0.36 | 1.84 | (2, 4) |
| *Pterodroma ultima* | Murphy's petrel | Pr | 927,188 | 32.40 | 6.04 | 31.31 | (2, 4, 25) |
| *Puffinus assimilis* | Little shearwater | Pr | 367,499 | 1.72 | 0.32 | 1.66 | (2, 4) |
| *Puffinus auricularis* | Newell’s shearwater | Pr | 765 | --- | --- | --- | (2, 4) |
| *Puffinus gavia* | Fluttering shearwater | Pr | 122,500 | 0.86 | 0.16 | 0.83 | (2, 4, 25) |
| *Puffinus heinrothi* | Heinroth's shearwater | Pr | 809 | --- | --- | --- | (2, 4) |
|  |  |  |  |  |  |  |  |

**Supplementary Table S1**. Cont.

|  |  |  |  |  |  |  |  |
| --- | --- | --- | --- | --- | --- | --- | --- |
| **Species** | **Common name** | **Order*** | **PBASC**** | **Total Cd**  **excreted** | **Total Pb**  **excreted** | **Total Hg excreted** | **Reference** |
|  |  |  |  |  |  |  |  |
|  |  |  |  |  |  |  |  |
|  |  |  |  | **(kg y^-1^)***** | | |  |
|  |  |  |  |  |  |  |  |
|  |  |  |  |  |  |  |  |
| *Puffinus huttoni* | Hutton's shearwater | Pr | 261,300 | 1.81 | 0.34 | 1.75 | (2, 4, 25) |
| *Puffinus lherminieri* | Audubon’s shearwater | Pr | 51,800 | 0.16 | 0.03 | 0.15 | (2, 4, 25) |
| *Puffinus mauretanicus* | Balearic shearwater | Pr | 24,225 | 0.21 | 0.04 | 0.20 | (2, 4) |
| *Puffinus nativitatis* | Christmas shearwater | Pr | 183,750 | 4.94 | 0.92 | 4.77 | (2, 4, 25) |
| *Puffinus opisthomelas* | Black-vented shearwater | Pr | 100,450 | 0.56 | 0.10 | 0.54 | (2, 4) |
| *Puffinus Puffinus* | Manx shearwater | Pr | 992,250 | 5.37 | 1.00 | 5.19 | (2, 4, 25) |
| *Puffinus yelkouan* | Yelkouan shearwater | Pr | 122,820 | 0.64 | 0.12 | 0.62 | (2, 4) |
| *Thalassarche bulleri* | Buller's albatross | Pr | 96,750 | 23.74 | 4.43 | 22.94 | (2-4) |
| *Thalassarche carteri* | Indian yellow-nosed albatross | Pr | 91,840 | 24.43 | 4.56 | 23.61 | (2-4) |
| *Thalassarche cauta* | Shy albatross | Pr | 41,445 | 14.07 | 2.62 | 13.59 | (2-4) |
| *Thalassarche chlororhynchos* | Atlantic Yellow-nosed albatross | Pr | 67,813 | 14.41 | 2.69 | 13.92 | (2-4) |
| *Thalassarche chrysostoma* | Grey-headed albatross | Pr | 350,000 | 58.66 | 10.94 | 56.67 | (2-4) |
| *Thalassarche eremita* | Chatham albatross | Pr | 13,750 | 4.70 | 0.88 | 4.54 | (2-4) |
| *Thalassarche impavida* | Campbell albatross | Pr | 54,336 | 14.85 | 2.77 | 14.35 | (2-4) |
| *Thalassarche melanophrys* | Black-browed albatross | Pr | 1,757,000 | 619.84 | 115.59 | 598.91 | (2-4) |
| *Thalassarche salvini* | Salvin's albatross | Pr | 99,988 | 33.94 | 6.33 | 32.79 | (2-4) |
| *Thalassarche steadi* | White-capped albatross | Pr | 251,250 | 87.00 | 16.23 | 84.07 | (2-4) |
| *Thalassoica antarctica* | Antarctic Petrel | Pr | 12,562,500 | 443.81 | 82.76 | 428.82 | (2, 4, 25) |
|  |  |  |  |  |  |  |  |
| **Total for this order:** |  | **Pr** | **391,010,783** | **8,472** | **1,580** | **8,186** |  |
|  |  |  |  |  |  |  |  |
| *Aptenodytes forsteri* | Emperor penguin | S | 776,475 | 481.99 | 219.63 | 77.33 | (2, 4, 32) |
| *Aptenodytes patagonicus* | King penguin | S | 5,329,700 | 1070.85 | 487.96 | 171.81 | (2, 4, 30) |
| *Eudyptes chrysocome* | Rockhopper penguin | S | 3,037,500 | 182.43 | 83.13 | 29.27 | (2, 4) |
| *Eudyptes chrysolophus* | Macaroni penguin | S | 15,372,000 | 1278.46 | 582.56 | 205.12 | (2, 4) |
| *Eudyptes pachyrhynchus* | Fiordland penguin | S | 9,375 | 0.73 | 0.33 | 0.12 | (2, 4) |
| *Eudyptes robustus* | Snares penguin | S | 83,160 | 6.62 | 3.02 | 1.06 | (2, 4) |
| *Eudyptes schlegeli* | Royal penguin | S | 2,074,000 | 209.82 | 95.61 | 33.66 | (2, 4) |
| *Eudyptes sclateri* | Erect-crested penguin | S | 198,000 | 17.11 | 7.80 | 2.75 | (2, 4) |
| *Eudyptula minor* | Little penguin | S | 551,968 | 12.19 | 5.56 | 1.96 | (2, 4, 31) |
| *Megadyptes antipodes* | Yellow-eyed penguin | S | 4,760 | 0.42 | 0.19 | 0.07 | (2, 4) |
| *Pygoscelis adeliae* | Adelie penguin | S | 13,173,433 | 959.08 | 437.03 | 153.88 | (2, 4) |
| *Pygoscelis antarcticus* | Chinstrap penguin | S | 12,800,000 | 990.24 | 451.23 | 158.88 | (2, 4) |
| *Pygoscelis papua* | Gentoo penguin | S | 1,161,000 | 93.97 | 42.82 | 15.08 | (2, 4) |
| *Spheniscus demersus* | African penguin | S | 65,250 | 2.70 | 1.23 | 0.43 | (2, 4) |
| *Spheniscus humboldti* | Humboldt penguin | S | 43,200 | 2.39 | 1.09 | 0.38 | (2, 4) |
| *Spheniscus magellanicus* | Magellanic penguin | S | 3,456,000 | 126.66 | 57.72 | 20.32 | (2, 4) |
| *Spheniscus mendiculus* | Galapagos penguin | S | 2,100 | 0.07 | 0.03 | 0.01 | (2, 4) |
|  |  |  |  |  |  |  |  |
| **Total for this order:** |  | **S** | **58,137,921** | **5,436** | **2,477** | **872** |  |
|  |  |  |  |  |  |  |  |
| **Total** |  |  | **745,289,756** | **39,328** | **27,247** | **35,676** |  |
|  |  |  |  |  |  |  |  |

*:Charadriiformes (C); Pelecaniformes (Pe); Procellariiformes (Pr); Sphenisciformes (S)

**: The number of chicks was calculated by applying a reproductive efficiency factor for each species, calculated by Riddick et al.^32^

***: Total excreted Cd, Pb and Hg are referred to breeders and chicks in the colony

**Supplementary Table** **S2**. F_E_ factor (theoretical content of each metal in g), obtained from the references and relative to the theoretical nitrogen content (0.036 g N g^-1^)^46^ for the different orders of birds and study elements.

|  |  |  |  |
| --- | --- | --- | --- |
| Order | **Pb** | **Cd** | **Hg** |
|  |  |  |  |
|  |  |  |  |
| Charadriiformes | 2.13x10^-4^ | 2.08x10^-4^ | 7.62x10^-6^ |
| Pelecaniformes | 2.00x10^-5^ | 7.36x10^-5^ | 5.12x10^-4^ |
| Procellariiformes | 1.40x10^-5^ | 7.50x10^-5^ | 7.25x10^-5^ |
| Sphenisciformes | 8.12x10^-6^ | 1.78x10^-5^ | 2.86x10^-6^ |
|  |  |  |  |

**Supplementary Table S3**. Bibliographic compilation of Cd, Hg and Pb content in faecal material of different seabirds. ---:no data

|  |  |  |  |  |  |
| --- | --- | --- | --- | --- | --- |
| **Order** | **Species** | **Pb** | **Cd** | **Hg** | **Reference** |
|  |  |  |  |  |  |
|  |  |  |  |  |  |
|  |  | **(mg kg^-1^)** | | |  |
|  |  |  |  |  |  |
|  |  |  |  |  |  |
| Charadriiformes | *Larus cachinans* | 39.9 | 5.80 | --- | *47* |
| Charadriiformes | *Larus cachinans* | --- | --- | 2.87 | *48* |
| Charadriiformes | *Larus michahellis* | 2.00 | 4.00 | 0.60 | *49* |
| Charadriiformes | *Larus michahellis* | 4.70 | 0.33 | 0.89 | *50* |
| Charadriiformes | *Larus michahellis* | 8.30 | 0.55 | --- | *51* |
| Charadriiformes | *Larus michahellis* | 5.96 | 9.06 | --- | *64* |
| Charadriiformes | *Larus dominicanus* | --- | 15.5 | --- | *52* |
| Charadriiformes | *Larus novaehollandiae* | --- | 37.7 | --- | *52* |
| Charadriiformes | *Sterna vittata* | --- | 62.8 | --- | *52* |
| Charadriiformes | *Stercorarius antarctica* | --- | 9.58 | --- | *52* |
| Charadriiformes | *Larus dominicanus* | --- | 54.1 | --- | *53* |
| Charadriiformes | *Stercorarius antarcticus* | --- | 2.23 | --- | *53* |
| Charadriiformes | *Larus marinus* | --- | 53.0 | --- | *54* |
| Charadriiformes | Larus fuscus | 5.2 | 0.1 | --- | *65* |
| Charadriiformes | Larus fuscus | 14.5 | 0.6 | --- | *65* |
| Charadriiformes | Larus fuscus | 11.8 | 0.3 | --- | *65* |
| Charadriiformes | Larus fuscus | 16.8 | 0.4 | --- | *65* |
| Charadriiformes | Larus fuscus | 27.6 | 0.5 | --- | *65* |
| Charadriiformes | Larus fuscus | 7.6 | 0.3 | --- | *65* |
| Charadriiformes | Larus fuscus | 16 | 0.7 | --- | *65* |
| Charadriiformes | Cerorhinca monocerata |  |  | 0.22 | *66* |
| Charadriiformes | Larus smithsoniansus |  |  | 0.15 | *67* |
| Charadriiformes | Larus smithsoniansus |  |  | 0.16 | *67* |
| Charadriiformes | Larus smithsoniansus |  |  | 0.1 | *67* |
| Charadriiformes | Larus smithsoniansus |  |  | 0.08 | *67* |
| Charadriiformes | Larus smithsoniansus |  |  | 0.095 | *67* |
| Charadriiformes | Larus smithsoniansus |  |  | 0.075 | *67* |
| Pelecaniformes | *Sula sula* | 1.60 | 6.34 | 108 | *55* |
| Pelecaniformes | *Sula sula* | 2.61 | 6.62 | --- | *56* |
| Pelecaniformes | *Sula sula* | --- | --- | 0.09 | *57* |
| Pelecaniformes | *Leucocarbo colensoi* | --- | 17.9 | --- | *53* |
| Pelecaniformes | *Stictocarbo punctatus* | --- | 0.20 | --- | *58* |
| Procellariiformes | *Fulmarus glacialis* | 2.46 | 8.40 | --- | *59* |
| Procellariiformes | *Ardenna grisea* | --- | 3.29 | --- | *52* |
| Procellariiformes | *Daption capense* | --- | 22.6 | --- | *52* |
| Procellariiformes | *Thalassarche bulleri* | --- | 3.29 | --- | *52* |
| Procellariiformes | *Diomedea epomophora* | --- | 5.75 | --- | *53* |
| Procellariiformes | *Diomedea exulans* | --- | 50.2 | --- | *53* |
| Procellariiformes | *Macronectes giganteus* | --- | 7.79 | --- | *53* |
| Procellariiformes | *Procellaria westlandica* | --- | 4.2 | --- | *58* |
| Sphenisciformes | *Pygoscelis papua ellsworthii* | 0.40 | 1.03 | --- | *60* |
| Sphenisciformes | *Pygoscelis papua* | 2.06 | 2.38 | --- | *61* |
|  |  |  |  |  |  |

**Supplementary Table S3**. Cont.

|  |  |  |  |  |  |
| --- | --- | --- | --- | --- | --- |
| **Order** | **Species** | **Pb** | **Cd** | **Hg** | **Reference** |
|  |  |  |  |  |  |
|  |  |  |  |  |  |
|  |  | **(mg kg^-1^)** | | |  |
|  |  |  |  |  |  |
|  |  |  |  |  |  |
| Sphenisciformes | *Pygoscelis antarctica* | 1.18 | 2.55 | --- | *61* |
| Sphenisciformes | *Pygoscelis Papua* | 0.83 | 1.93 | --- | *62* |
| Sphenisciformes | *Eudyptes robustus* | --- | 23.7 | --- | *52* |
| Sphenisciformes | *Megadyptes antipodes* | --- | 7.38 | --- | *53* |
| Sphenisciformes | *Eudyptula minor* | 0.92 | 0.45 | 0.25 | *63* |
| Sphenisciformes | *Pygoscelis adeliae* | --- | 0.90 | --- | *54* |
| Sphenisciformes |  | 0,43 | 5,8 | 0,34 | *68* |
| Sphenisciformes |  | --- | --- | 0,15 | *68* |
| Sphenisciformes |  |  |  | 0,17 | *68* |
| Sphenisciformes |  | 3,8 | 0,16 |  | *68* |
| Sphenisciformes |  | --- | --- | 1,6 | *68* |
| Sphenisciformes | Pygoscelis Papua | 0,1 | 1,6 | --- | *68* |
| Sphenisciformes | Pygoscelis Papua | 0,1 | 1,2 | --- | *68* |
| Sphenisciformes | Pygoscelis Papua | 1,5 | 2 | --- | *68* |
| Sphenisciformes | Pygoscelis Papua | 1,7 | 2,9 | --- | *68* |
| Sphenisciformes | Pygoscelis adeliae | 2 | 4 | 0,52 | *68* |
| Sphenisciformes | Pygoscelis adeliae | 1,5 | 2,8 | 0,4 | *68* |
| Sphenisciformes | Pygoscelis adeliae | 0,58 | 1,8 | 0,1 | *68* |
| Sphenisciformes | Pygoscelis adeliae | 0,44 | 1,6 | 0,13 | *68* |
| Sphenisciformes |  | 2,1 | --- | 0,2 | *68* |
| Sphenisciformes |  | 1,4 | --- | 0,11 | *68* |
| Sphenisciformes |  | 0,11 | --- | 0,15 | *68* |
| Sphenisciformes |  | 0,14 | 1,3 | --- | *68* |
| Sphenisciformes |  | 0,15 | 0,92 | --- | *68* |
| Sphenisciformes |  | 0,19 | 2,4 | --- | *68* |
| Sphenisciformes |  | 0,1 | 1,1 | --- | *68* |
| Sphenisciformes |  | 0,22 | 1,3 | --- | *68* |
| Sphenisciformes |  |  | 1,6 | --- | *68* |
| Sphenisciformes |  | 1 | 2 | 0,035 | *69* |
|  |  |  |  |  |  |

**Supplementary Table S4**. Excretion of Cd, Hg and Pb by the five species (adults and chicks) that contribute most to the amounts excreted globally

.

|  |  |  |  |
| --- | --- | --- | --- |
| **Species** | **Common name** | **Element** | **Excretion**  **(kg y^-1^)** |
|  |  |  |  |
|  |  |  |  |
| *Adult seabirds* | | | |
|  |  |  |  |
| *Uria aalge* | Common Guillemot | Cd | 3311 |
| *Uria lomvia* | Thick-billed Murre | Cd | 3131 |
| *Fulmarus glacialis* | Northern Fulmar | Cd | 1394 |
| *Ardenna tenuirostris* | Short-tailed Shearwater | Cd | 1260 |
| *Eudyptes chrysolophus* | Macaroni Penguin | Cd | 1048 |
|  |  |  |  |
| *Phalacrocorax bougainvilliorum* | Guanay Cormorant | Hg | 3345 |
| *Morus bassanus* | Northern Gannet | Hg | 2933 |
| *Fulmarus glacialis* | Northern Fulmar | Hg | 1347 |
| *Ardenna tenuirostris* | Short-tailed Shearwater | Hg | 1218 |
| *Sula variegata* | Peruvian booby | Hg | 1092 |
|  |  |  |  |
| *Uria aalge* | Common Guillemot | Pb | 3384 |
| *Uria lomvia* | Thick-billed Murre | Pb | 3200 |
| *Alle alle* | Little Auk | Pb | 1004 |
| *Rissa tridactyla* | Black-legged Kittiwake | Pb | 934 |
| *Onychoprion fuscatus* | Sooty Tern | Pb | 670 |
|  |  |  |  |
| *Chicks* | | | |
|  |  |  |  |
| *Uria lomvia* | Thick-billed Murre | Cd | 1252 |
| *Uria aalge* | Common Guillemot | Cd | 1175 |
| *Phalacrocorax bougainvilliorum* | Guanay Cormorant | Cd | 577 |
| *Fulmarus glacialis* | Northern Fulmar | Cd | 397 |
| *Ardenna tenuirostris* | Short-tailed Shearwater | Cd | 378 |
|  |  |  |  |
| *Phalacrocorax bougainvilliorum* | Guanay Cormorant | Hg | 4014 |
| *Morus bassanus* | Northern Gannet | Hg | 1071 |
| *Phalacrocorax carbo* | Great Cormorant | Hg | 976 |
| *Phalacrocorax brasilianus* | Neotropic Cormorant | Hg | 649 |
| *Phalacrocorax atriceps* | Imperial Shag | Hg | 617 |
|  |  |  |  |
| *Uria lomvia* | Thick-billed Murre | Pb | 1280 |
| *Uria aalge* | Common Guillemot | Pb | 1201 |
| *Rissa tridactyla* | Black-legged Kittiwake | Pb | 364 |
| *Alle alle* | Little Auk | Pb | 251 |
| *Larus dominicanus* | Kelp Gull | Pb | 248 |
|  |  |  |  |

**Supplementary Table S5.** Global natural and anthropogenic emissions and fluxes of Cd between different geochemical compartments.

|  |  |  |
| --- | --- | --- |
| **Compartment** | **Cd flux**  **(Mg y^-1^)** | **Reference** |
|  |  |  |
|  |  |  |
| *Natural* | | |
|  |  |  |
| Volcanoes | 270-820 | *33-35,71* |
| Volcanic dust flux | 40 | *37* |
| Volcanic gas flux | 0.1 | *37* |
| Biogenic | 200-240 | *34, 35* |
| Eolian | 200-210 | *34, 35,71* |
| Terrestrial biomass burning | 110 | *35* |
| Wildfires | 10-100 | *33, 34* |
| Sea-salt spray | 2-100 | *33-35* |
| Riverine flux | 2352-3000 | *36,71* |
| Natural annual emissions | 300-1000 | *33* |
| Manmade annual emissions | 6,000-7,000 | *33* |
| Continental dust flux | 250 | *37* |
| Atmospheric dust | 250 | *33* |
|  |  |  |
| *Anthropogenic* | | |
|  |  |  |
| Fossil fuel combustion | 691-800 | *34, 35* |
| Non-ferrous metal production | 2171 | *35* |
| Cement production | 64 | *35* |
| Waste disposal | 40-800 | *34, 35* |
| Industry | 6,000 | *34* |
| Industrial particulate emissions | 4,000 | *37* |
| Fossil Fuel Flux | 1,500 | *37* |
| Total emissions: industrial plus fossil fuel | 5,500 | *37* |
|  |  |  |
| *Transfer of Cd from surface ocean to land by seabirds* | | |
|  |  |  |
| Breeding seabirds | 39 | This study |
| Total population | 249 | This study |
|  |  |  |

**Supplementary Table S6**. Major compartments of Hg in the Earth’s surface and fluxes between different geochemical compartments.

|  |  | |  |
| --- | --- | --- | --- |
| **Compartment** | **Value**  **(Mg y^-1^)** | | **Reference** |
|  |  | |  |
|  |  | |  |
|  |  | |  |
| *Natural Hg fluxes* | | | |
|  |  | |  |
| Volcanoes | 30-1,000 | | *33, 34,71* |
| Volcanic dust flux | 10 | | 37  37 |
| Volcanic gas flux | 0.1 | |  |
| Biogenic | 1,400 | | *34* |
| Riverine flux | | 1,440 | *71* |
| Eolian | 100 | | *34* |
| Wildfires | 100-600 | | *33,70,71*  *33,71*  *33*  *33* |
| Sea-salt spray | 3-9 | |  |
| Natural annual emissions | 200-25,000 | |  |
| Manmade annual emissions | 11,000 | |  |
| Continental dust flux | 30 | | *37* |
| Atmospheric dust | 30 | | 37 |
|  |  | |  |
| *Anthropogenic Hg fluxes* | | | |
|  |  | |  |
| Fossil fuel combustion | 2,300 | | *34*  *34*  *34*  *37*  *37*  *37* |
| Waste disposal | 1,200 | |  |
| Industry | 100 | |  |
| Industrial particulate emissions | 5,000 | |  |
| Fossil fuel flux | 6,000 | |  |
| Total emissions: industrial plus fossil fuel | 11,000 | |  |
|  |  | |  |
| *Fluxes of Hg among compartments* | | | |
|  |  | |  |
| Land soil → atmosphere | 1,000-2,800 | | *39-44* |
| Geogenic → atmosphere | 500 | | *39*  *39*  *39* |
| Anthropogenic → atmosphere | 3,400 | |  |
| Biomass burning → atmosphere | 600 | |  |
| Surface ocean → atmosphere | 800-5,600 | | *39-44* |
| Atmosphere → land soil | 2,200-5,000 | | *39-44* |
| Atmosphere → surface ocean | 2,000-7,100 | | *39-44* |
| Soil → surface ocean | 200-380 | | *39-44* |
| Surface ocean → deep ocean | 4,100 | | *39*  *39* |
| Deep ocean → surface ocean | 1,800 | |  |
| Deep ocean → sediment | 200-600 | | *39-44* |
|  |  | |  |
| *Fluxes of Hg from surface ocean to land by seabirds (Mg y^-1^)* | | | |
|  |  | |  |
| Breeding seabirds | 36 | | This study  This study |
| Total seabird population | 226 | |  |
|  |  | |  |

**Supplementary Table S7.** Global natural and anthropogenic emissions and fluxes of Pb between different geochemical compartments.

|  |  |  |
| --- | --- | --- |
| **Compartment** | **Pb flux**  **(Mg y^-1^)** | **Reference** |
|  |  |  |
|  |  |  |
| *Natural* | | |
|  |  |  |
| Volcanoes | 860 | *71* |
| Biogenic | 1,700-1,740 | *34, 45* |
| Eolian | 3,900-162,000 | *34, 45,71* |
| Terrestrial biomass burning | 1,900 | *45* |
| Wildfires | 500-1,900 | *33, 34* |
| Sea-salt spray | 10 | *71* |
| Riverine flux | 3,000-408,000 | *36,71* |
| Natural annual emissions | 4,000-19,000 | *33* |
| Manmade annual emissions | 450,000 | *33* |
| Continental dust flux | 5,000 | *37* |
| Volcanic dust flux | 870 | *37* |
| Volcanic gas flux | 1.2 | *37* |
| Atmospheric dust | 10,000 | *33* |
|  |  |  |
| *Anthropogenic* | | |
|  |  |  |
| Fossil fuel combustion | 11,690-12,700 | *34, 45* |
| Vehicular traffic | 88,739 | *45* |
| Non-ferrous metal production | 14,815 | *45* |
| Iron and steel production | 2,926 | *45* |
| Cement production | 268 | *45* |
| Waste disposal | 821-2,400 | *34, 45* |
| Industry | 62,200 | *34* |
| Mining industry | 2,600 | *34* |
| National and international trade | 252,500 | *34* |
| Industrial particulate emissions | 1,600,000 | *37* |
| Fossil Fuel Flux | 430,000 | *37* |
| Total emissions: industrial plus fossil fuel | 2,030,000 | *37* |
|  |  |  |
| *Transfer of Pb from surface ocean to land by seabirds* | | |
|  |  |  |
| Breeding seabirds | 27 | This study |
| Total population | 172 | This study |
|  |  |  |

**Supplementary Table S8**. Total concentration and metal partitioning (% with respect to total metal content) in faecal material of Yellow-legged gull (*Larus michahellis*) in colonies from NW Spain.

|  |  |  |  |  |
| --- | --- | --- | --- | --- |
| **Metal** | **Exchangeable and carbonate**  **(µg kg^-1^)** | **Reducible**  **(µg kg^-1^)** | **Oxidizable**  **(µg kg^-1^)** | **Metal Total concentration**  **(µg kg^-1^)** |
|  |  |  |  |  |
|  |  |  |  |  |
| Cd | (7.0±5.1)x10^2^ (13.7) | (5.3±4.0)x10^2^ (10.4) | (3.3±3.3)x10^2^ (6.40) | (51±42)x10^2^ |
| Hg | 2.5±3.7 (3) | <0.50 | 23±8.3(31) | 82±42 |
| Pb | 66±43 (12.8) | 38±19 (7.48) | 70±42 (13.7) | (5.1±4.5)x10^2^ |
|  |  |  |  |  |

**References supplementary Tables S1 to S8**

1. BirdLife International (BI). *Threatened birds of Asia: the BI Red Data Book*. (BI, Cambridge, UK 2001).
2. BirdLife International (BI) IUCN Red List for birds. http://www.birdlife.org (2019).
3. del Hoyo, J., Elliott, A. & Sargatal, J. *Handbook of the Birds of the World*, vol. 3: Hoatzin to Auks (Lynx Edicions, Spain, 1996).
4. Croxall, J. P. et al. Seabird conservation status, threats and priority actions: a global assessment. *Bird Conserv. Int.* **22**, 1–34 (2012). <https://doi.org/10.1017/S0959270912000020>
5. Joint Nature Conservation Committee (JNCC) (<http://www.jncc.defra.gov.uk>, (2019).
6. Wetlands International. *Waterbird Population Estimates*. (4th ed. Wetlands International, Wageningen, The Netherlands, 2006).
7. U.S. Fish and Wildlife Service. *Endangered and Threatened Wildlife and Plants; Review of Native Species That Are Candidates for Listing as Endangered or Threatened*. (<http://www.fws.gov/endangered/what-we-do/cnor.html>, 2016).
8. COSEWIC. Update COSEWIC Status Report on the Marbled Murrelet (*Brachyramphus marmoratus*) in Canada. (Committee on the Status of Wildlife In Canada, Ottawa, ON 2012).
9. John, R. C. et al. *Ecology and conservation of the Marbled Murrelet*. (Gen. Tech. Rep. PSW-GTR-152, Pacific Southwest Research Station, Forest Service, U.S. Department of Agriculture, 1995).
10. Castro, D. et al. Aves, Stercorariidae, Chilean Skua *Stercorarius chilensis* Bonaparte, 1857: First documented record for the state of Espírito Santo, Southeastern Brazil. *Check List* **8**, 560–562 (2012). <https://doi.org/10.15560/8.3.560>
11. Department of the Environment. *Stercorarius antarcticus lonnbergi* in Species Profile and Threats Database (online database). Available at: <http://www.environment.gov.au/sprat> (2020).
12. Fang, E. D. Snowy Sheathbill (*Chionis albus*). in *Neotropical Birds Online* (ed Schulenberg, T.S.) <http://neotropical.birds.cornell.edu/portal/species/overview> (2010)
13. The MarineBio Conservation Society. <http://marinebio.org/membership/join> (2020).
14. Suárez, N., Retana, M. V. & Yorio, P. Spatial patterns in the use of foraging areas and its relationship with prey resources in the threatened Olrog’s Gull (*Larus atlanticus*). *J. Ornithol.* **153**, 861–871 (2012). <https://doi.org/10.1007/s10336-012-0812-8>
15. Yorio, P., Quintana, F., Gatto, A., Lisnizer, N. & Suárez, N. Foraging patterns of breeding Olrog's Gull at Golfo San Jorge, Argentina. *Waterbirds* **27**, 193–199 (2004). [https://doi.org/10.1675/1524-4695(2004)027[0193:FPOBOG]2.0.CO;2](https://doi.org/10.1675/1524-4695(2004)027%5b0193:FPOBOG%5d2.0.CO;2)
16. Kushlan, J. A. et al. *Waterbird conservation for the Americas*. (U.S. Fish & Wildlife Service National Publications Clearinghouse, Shepherdstown, U.S.A., 2002).
17. Harfenist, A. Cassin’s auklet (*Ptychoramphus aleuticus*) (Accounts and Measures for Managing Identified Wildlife-Accounts, British Columbia, Canada, 2004).
18. Wetlands International, *Waterbird Population Estimates*. <http://wpe.wetlands.org/> (2020).
19. Encyclopedia of Life. <http://www.eol.org> (2020).
20. Birt, T. et al. Rangewide population genetic structure of Xantus´s Murrelet (*Synthliboramphus hypoleucus*). *Auk* **129**, 44–55 (2012). <https://doi.org/10.1525/auk.2011.11011>
21. Hill, R. & Dunn, A. National recovery plan for the Christmas Island Frigatebird (*Fregata andrewsi*) (Commonwealth of Australia, Canberra. 2004).
22. Bunce, A., Norman, F., Brothers, N. & Gales, R. Long-term trends in the Australasian gannet (*Morus serrator*) population in Australia: the effect of climate change and commercial fisheries. *Mar. Biol.* **141**, 263–269 (2002). <https://doi.org/10.1007/s00227-002-0838-1>
23. Jennings, M. C. Atlas of the breeding birds of Arabia. *Fauna Arabia* **25**, 1–772 (2010
24. Animal Diversity, *Sula nebouxii*, blue-footed booby (Online). Available at: <https://animaldiversity.org/accounts/Sula_nebouxii/> (cited 22 March 2020).
25. Brooke, M. L. *Albatrosses and petrels across the world* (University Press, Oxford, 2004).
26. Marchant, S. & Higgins, P. J. *Handbook of Australian, New Zealand and Antarctic Birds, 1: ratites to ducks* (Oxford University Press, Oxford, UK,1990).
27. Luigi, G., Bugoni, L., Fonseca-Neto, F. P. & Teixeira, D. M. Biologia e conservação do petrel-de trindade, *Pterodroma arminjoniana*, na ilha da Trindade, Atlântico sul. In*: Ilhas oceánicas brasileiras: da pesquisa ao manejo* (eds Mohr, L. V., Castro, J. W. A., Costa, P. M. S. & Alves, R. J. V.) vol 2 (Ministério do Meio Ambiente, Brasília, Brazil, 2008).
28. Brooke, M. L. et al. The potential for rat predation to cause decline of the globally threatened Henderson petrel *Pterodroma atrata*: evidence from population modelling, the field and stable isotopes. *Endanger. Species Res.* **11**, 47–59 (2010). <https://doi.org/10.3354/esr00249>
29. Scott, D. et. al. Predictive habitat modelling to estimate petrel breeding colony sizes: Sooty shearwaters (*Puffinus griseus*) and mottled petrels (*Pterodroma inexpectata*) on Whenua Hou Island. *N.Z. J. Zool.* **36**, 291–306 (2009). <https://doi.org/10.1080/03014220909510156>
30. Shirihai, H. A. *Complete Guide to Antarctic Wildlife* (A&C Black, London, UK, 2002).
31. del Hoyo, J., Elliot, A. & Sargatal, J. *Handbook of the Birds of the World, vol. 1: Ostrich to Ducks* (Lynx Edicions, Barcelona, 1992).
32. Riddick, S. N. et al. The global distribution of ammonia emissions from seabird colonies. *Atmos. Environ.* **55**, 319–327 (2012). <https://doi.org/10.1016/j.atmosenv.2012.02.052>
33. Merian, E. (Ed.) *Metals and Their Compounds in the Environment. Occurrence, Analysis and Biological Relevance* (Wiley-Blackwell, New Jersey, 1991).
34. Macías, F. & Calvo de Anta, R. *Niveles genéricos de referencia de metales pesados y otros elementos traza en los suelos de Galicia* (Xunta de Galicia, Spain, 2009).
35. Cullen, J. & Maldonado, M. Biogeochemistry of cadmium and its release to the environment. *Met. Ions Life Sci.* **11**, 31–62 (2013). <https://doi.org/10.1007/978-94-007-5179-8_2>
36. Gaillardet, J., Viers, J., Dupré, B. in *Treatise on Geochemistry,* vol. 5 (eds Holland, H. D. & Turekian, K. K.) 225–272 (Elsevier-Pergamon, Oxford, 2003).
37. Morgan, J. J. & Stumm, W. in *Metals and Their Compounds in the Environment. Occurrence, Analysis and Biological Relevance* (eds Merian, E., Anke, M., Ihnat, M. & Stoeppler, M.) 67–104 (VCH Publishers, Germany, 1991).
38. Anderson, W. B. & Polis, G. A. in *Food Web at the Landscape Level* (eds. Polis, G. A., Power, M. E. & Huxel, G. R.) 82-95 (University of Chicago Press, Illinois, 2004).
39. Selin, N. E. Global biogeochemical cycling of mercury: A review. *Annu. Rev. Environ. Resour.* **34(1)**, 43–63 (2009). <https://doi.org/10.1146/annurev.environ.051308.084314>
40. Seline, N. E. et al., Global 3-D land-ocean-atmosphere model for mercury: present-day versus preindustrial cycles and anthropogenic enrichment factors for deposition. *Glob. Biogeochem. Cycles* **22**, GB2011 (2008). <https://doi.org/10.1029/2007GB003040>
41. Sunderland, E. M. & Mason, R. P. Human impacts on open ocean mercury concentrations. *Glob. Biogeochem. Cycles* **21**, GB4022 (2007). <https://doi.org/10.1029/2006GB002876>
42. Mason, R. P., Fitzgerald, W. F., Morel, F. M. M. The biogeochemical cycling of elemental mercury: anthropogenic influences. *Geochim. Cosmochim. Acta* **58**, 3191–3198 (1994). <https://doi.org/10.1016/0016-7037(94)90046-9>
43. Lamborg, C. H. et al. Modern and historic atmospheric mercury fluxes in both hemispheres: global and regional mercury cycling implications. *Glob. Biogeochem. Cycles* **16**, 1104–1114 (2002). <https://doi.org/10.1029/2001GB001847>
44. Mason, R. P. & Sheu, G-R. Role of the ocean in the global mercury cycle. *Glob. Biogeochem. Cycles* **16**, 1093 (2002). <https://doi.org/10.1029/2001GB001440>
45. Cullen, J. & McAlister, J. “Biogeochemistry of Lead. Its Release to the Environment and Chemical Speciation” in *Lead: Its Effects on Environment and Health* (De Gruyter, Berlin, Boston, 2017), pp. 21–48.
46. Furness, R. W. “The occurrence of burrow-nesting among birds and its influence on soil fertility and stability” in *Symposia of the Zoological Society of London; The Environmental Impact of Burrowing Animals and Animal Burrows* (Zoological Society of London, UK, 1991), pp. 53–67.
47. Otero, X.L. Effects of nesting yellow-legged gulls (*Larus cachinnans* Pallas) on the heavy metals content of soils in Cíes Islands (Galicia-NW Spain). *Mar. Pollut. Bull.* **36**, 267–272 (1998). <https://doi.org/10.1016/S0025-326X(98)80010-6>
48. Otero, X. L. & Mouriño, J. Nitrógeno (NH_4_^+^, NO_3_^-^), Fósforo asimilable y metales traza (Hg, Cd, Pb, Zn, Ni y Cu) en suelos de la colonia de la gaviota patiamarilla (Larus cachinnans) en el Parque natural de las Islas Cíes. *Cuad. Soc. Esp. Cien. For.* **14**, 143–149 (2002). <https://doi.org/10.31167/csef.v0i14.9311>
49. De La Peña-Lastra, S., Otero, X. L. & Pérez-Alberti, A. Enrichment of trace elements in colonies of the yellow-legged gull (*Larus michahellis*) in the Atlantic Islands National Park (Galicia-NW Spain). *Sci. Total Environ.* **648**, 1536–1548 (2019). <https://doi.org/10.1016/j.scitotenv.2018.08.284>
50. Signa, G., Mazzola, A., Tramati, C. D. & Vizzini, S. Gull-derived trace elements trigger small-scale contamination in a remote Mediterranean nature reserve. *Mar. Pollut. Bull.* **74**, 237–243 (2013). <https://doi.org/10.1016/j.marpolbul.2013.06.051>
51. Rial, D. et al. Toxicity of seabird guano to sea urchin embryos and interaction with Cu and Pb. *Chemosphere* **145**, 384–393 (2016). <https://doi.org/10.1016/j.chemosphere.2015.11.064>
52. Wing, S. R., Wing, L. C., Shatova, O. A. & Van Hale, R. Marine micronutrient vectors: seabirds, marine mammals and fishes egest high concentrations of bioactive metals in the subantarctic island ecosystem. *Mar. Ecol. Prog. Ser.* **563**, 13–23 (2017). <https://doi.org/10.3354/meps11978>
53. Wing, S. R. et al. Seabirds and marine mammals redistribute bioavailable iron in the Southern Ocean. *Mar. Ecol. Prog. Ser.* **510**, 1−13 (2014). <https://doi.org/10.3354/meps10923>
54. Shatova, O. A., Wing, S.R., Hoffmann, L.J., Wing, L.C. & Gault-Ringold, M. Phytoplankton community structure is influenced by seabird guano enrichment in the Southern Ocean. *Estuar. Coast. Shelf Sci.* **191**, 125−135 (2017). <https://doi.org/10.1016/j.ecss.2017.04.021>
55. Liu, X. et al. P and trace metal contents in biomaterials, soils, sediments and plants in colony of red-footed booby (*Sula sula*) in the Dongdao Island of South China Sea. *Chemosphere* **65**, 707−715 (2006). <https://doi.org/10.1016/j.chemosphere.2006.01.043>
56. Yan, H. et al. A 2000-year record of copper pollution in South China Sea derived from seabird excrements: a potential indicator for copper production and civilization of China. *J. Paleolimnol.* **44**, 431–442 (2010). <https://doi.org/10.1007/s10933-010-9413-9>
57. Yan, H., Yuhong, W., Wenhan, C. & Liguang, S. Millennial mercury records derived from ornithogenic sediment on Dongdao Island, South China Sea. *J. Environ. Sci.* **23**, 1415–1423 (2011). <https://doi.org/10.1016/S1001-0742(10)60603-1>
58. Zhong, H., Kim, Y.-N., Smith, C., Robinson, B. & Dickinson, N. Seabird guano and phosphorus fractionation in a rhizosphere with earthworms. *Appl. Soil Ecol.* **120**, 197–205 (2017). <https://doi.org/10.1016/j.apsoil.2017.08.006>
59. Brimble, S. K. et al. High arctic ponds receiving biotransported nutrients from a nearby seabird colony are also subject to potentially toxic loadings of arsenic, cadmium and zinc. *Environ. Toxicol. Chem.* **28**, 2426–2433 (2009). <https://doi.org/10.1897/09-235.1>
60. Metcheva, R., Yurukova, L. & Teodorova, S. E. Biogenic and toxic elements in feathers, eggs, and excreta of Gentoo penguin (Pygoscelis papua ellsworthii) in the Antarctic. *Environ. Monit. Assess.* **182**, 571–585 (2011). <https://doi.org/10.1007/s10661-011-1898-9>
61. Espejo, W., Celis, J. E., González-Acuña, D., Jara, S. & Barra, R. Concentration of trace metals in excrements of two species of penguins from different locations of the Antarctic Peninsula. *Polar Biol.* **37**, 675–683 (2014). <https://doi.org/10.1007/s00300-014-1468-z>
62. Celis, J. E., Barra, R., Espejo, W., González-Acuña, D. & Jara, S. Trace element concentrations in biotic matrices of gentoo penguins (*Pygoscelis Papua*) and coastal soils from different locations of the Antarctic Peninsula. *Water Air Soil Pollut.* **226**, 2266 (2014). <https://doi.org/10.1007/s11270-014-2266-5>
63. Finger, A. et al. Metals and metalloids in Little Penguin (*Eudyptula minor*) prey, blood and faeces. *Environ. Pollut.* **223**, 567–574 (2017). <https://doi.org/10.1016/j.envpol.2017.01.059>
64. Alba-González, P., Álvarez-Salgado, X. A., Cobelo-García, A., Kaal, J., Teira, E. Faeces of marine birds and mammals as substrates for microbial plankton communities. *Mar. Environ. Res.* **174**, 105560 (2022). <https://doi.org/10.1016/j.marenvres.2022.105560>
65. Martín-Vélez, V., Hortas, F., Taggart, M. A., Green, A. J., ÓHanlon, N. J., Sánchez, M. I. Spatial variation and biovectoring of metals in gull faeces. *Ecol. Indic.* **125**,107534 (2021). <https://doi.org/10.1016/j.ecolind.2021.107534>
66. Shoji, A., Elliot, K. H., Aris-Brosou, S., Mizukawa, H., Nakayama, S. M. M., Ikenaka, Y., Ishizuka, M., Kuwae, T., Watanabe, K., Escoruela Gonzalez, J., Watanuki, Y. Biotransport of metallic trace elements from marine to terrestrial ecosystems by seabirds. *Environ. Toxicol. Chem.* **38**(1), 106–114 (2019). <https://doi.org/10.1002/etc.4286>
67. Geizer, H. D., Klapstein, S. J., Mallory, M. L., O'Driscoll, N. J. Total mercury, methylmercury, phosphate, and sulfate inputs to a bog ecosystem from herring gull (Larus smithsoniansus) guano. *Ecotoxicol. Environ. Saf.* **226**, 112845 (2021). <https://doi.org/10.1016/j.ecoenv.2021.112845>
68. Sparaventi, E., Rodríguez-Romero, A., Barbosa, A., Ramajo, L., Tovar-Sánchez, A. Trace elements in Antarctic penguins and the potential role of guano as source of recycled metals in the Southern Ocean. *Chemosphere* **285**, 131423 (2021). <https://doi.org/10.1016/j.chemosphere.2021.131423>
69. Chu, Z., Yang, Z., Wang, Y., Sun, L.,Yang, W., Yang, L., Gao, Y. Assessment of heavy metal contamination from penguins and anthropogenic activities on Fildes Peninsula and Ardley Island, Antarctic. *Sci Total Environ.* **646**, 951–957 (2019). <https://doi.org/10.1016/j.scitotenv.2018.07.152>
70. Kumara, A. et al. Mercury from wildfires. Global emission inventories and sensitivity to 2000–2050 global change. *Atmospheric Environment* **173**, (2018), 6-15, <https://doi.org/10.1016/j.atmosenv.2017.10.061>
71. Sen, I., Peucker-Ehrenbrink, B. Anthropogenic Disturbance of Element Cycles at the Earth's Surface. Environmental Science & Technology. 46. 2012. 8601-9. 10.1021/es301261x.
